# Healthy working life expectancy across birth cohorts in the United States

**DOI:** 10.1093/geronb/gbaf119

**Published:** 2025-06-27

**Authors:** Félix Blain, Michaël Boissonneault

**Affiliations:** Department of Demography, University of Montreal, Montreal, Quebec, Canada; Department of Demography, University of Montreal, Montreal, Quebec, Canada

**Keywords:** active life expectancy, population aging, employment, education, multistate model

## Abstract

**Objectives:**

This study investigates trends in healthy working life expectancy (HWLE) in the United States amid changing retirement conditions and recent declines in health among working-age individuals. HWLE, defined as the average number of years expected to be spent healthy and working between ages 51 and 80, is examined by gender and educational level across three birth cohorts.

**Methods:**

Using longitudinal data from the Health and Retirement Study (HRS), HWLE estimates were calculated for individuals aged 51 and older. Using continuous-time multistate modelling, trends were analyzed across three birth cohorts (1936–1941, 1942–1947, 1948–1953), focusing on differences by gender and educational attainment to assess disparities in HWLE over time.

**Results:**

The findings indicate that HWLE remained stable for most groups but declined among individuals with lower educational attainment. Thus, as early retirement becomes increasingly costly and risky, workers appear unable to extend their working lives and are facing growing inequalities.

**Discussion:**

These findings highlight the need for targeted policies to promote healthier work environments and expanded job opportunities for older adults. Addressing disparities in HWLE, particularly for those with less education, is critical to improving outcomes for future cohorts as they approach retirement age.

With the entry of the first cohorts of the baby boom into old age, several developed countries are experiencing accelerated population aging. To mitigate the pressure this places on pension and healthcare systems, several countries have passed laws that encourage delayed retirement among workers, including increases to the retirement age, or are planning to do so in the near future (OECD, 2023). Older workers thus face significant pressure to remain in the labor market longer. However, the ability of older individuals to stay employed while maintaining good health remains uncertain (Berger et al., 2022; Berkman & Truesdale, 2022).

In the United States, several factors have contributed to increasing the effective retirement age during the second half of the 1990s and the first half of the 2000s. These include greater female labor force participation, more educated younger cohorts, a decline in employer provision of retiree health insurance, and the shift from defined benefits to defined contribution plans among employers (Blau & Goodstein, 2010; Hurd & Rohwedder, 2011; Monk & Munnell, 2009; Schirle, 2008). They also include legislative changes such as the introduction of delayed retirement credits and the increase in the normal retirement age (from 65 to 66 among the cohorts born between 1937 and 1944) (Gustman & Steinmeier, 2009; Svahn & Ross, 1983). Taken together, these factors have contributed to increasing the effective retirement age by about one year for men and 2 years for women between 1995 and 2007 (Munnell, 2022).

However, although the same changes are still being phased in, average retirement ages have ceased to increase since the mid-2010s. Notably, the 2008 financial crisis and its aftermath have brought significant financial uncertainty among workers (Rhee, 2013). While this may have prompted some to delay retirement, the crisis also had the effect of limiting job opportunities, thus forcing many others into early, involuntary retirement (Coile & Levine, 2011). Thus, while the financial crisis undeniably modified the retirement landscape during the past decade, its effect on the age at which people retire is unclear.

Another trend we have been witnessing over the past decade is the historic decline in life expectancy (LE) among the American population, which was driven notably by an unprecedented increase in mortality among working-age individuals (Harper et al., 2021; Venkataramani et al., 2021). These trends are mirrored in the health profiles of younger cohorts in the United States, who exhibit higher rates of disability and chronic disease, as well as poorer self-reported health than those of preceding cohorts (King et al., 2013).

Meanwhile, the stagnation in the average retirement ages and worsening health outcomes have coincided with a strong increase in the intention to work longer: while 23% of workers aged 53–58 planned to continue working in 1994, this was the case for 41% in 2012 (Dong et al., 2017). Thus, there are strong indications that older workers in the United States feel pressure to extend their working lives. Nonetheless, the hurdle toward extending working lives has probably never been this high given the population’s current morbidity profile.

In this context, researchers have highlighted the need for continued monitoring of the employment, health, and longevity conditions of populations (Dudel, 2021; Head & Hyde, 2020; Nygård & Rantanen, 2017). Healthy working life expectancy (HWLE) is a measure that has proven effective in meeting this need (Lièvre et al., 2007, 2009). HWLE represents the cumulative average number of years that people can expect to be both healthy and in work from a given age. Thus, in this study, we aimed to estimate trends in HWLE from age 51 by gender and educational level across three birth cohorts in the United States.

Since 2020, several studies have measured HWLE in various contexts using different health measures (Hambisa et al., 2023; Heller et al., 2022; Li et al., 2024; Parker et al., 2020). Despite some variation, HWLE was lower than the remaining years to the pension age in almost all the countries investigated. Studies that have investigated trends in healthy and unhealthy working life expectancy (UHWLE) have reported increases in HWLE over time but also increases in UHWLE (Boissonneault & Ríos, 2021; Laaksonen et al., 2022; Lynch et al., 2022; Sperlich et al., 2023). Assessing trends in 14 Organization for Economic Co-operation and Development (OECD) countries, one study found increases in HWLE over time in all countries except one, the United States (Boissonneault & Ríos, 2021). This study is the only one so far that has provided estimates for the United States, but it has done so from a period perspective, i.e., basing its calculations on people of different ages at one point in time rather than following the same cohort of people over time across years of age. Also, most studies on HWLE only disaggregated results by gender.

Thus, estimates of HWLE have still not been produced concerning the United States that reflect the experience of entire cohorts of workers or that have accounted for variation between educational groups. The cohort-based approach is particularly relevant in the U.S. context, given that it is with reference to workers’ year of birth that different morbidity profiles have begun to emerge in recent decades and that many of the changes affecting the retirement landscape have been phased in. As such, it has the potential to shed light on the extent to which poor health may have formed an impediment toward the realization of longer working lives among American workers during the last decades.

## Method

### Study design and participants

Data comes from the Health and Retirement Study (HRS, 2024), a longitudinal panel study representative of the older population in the United States. The HRS survey has been conducted every 2 years since its inception in 1992. This survey follows a birth cohort sampling plan, where a new cohort is added to the initial sample every 6 years. The target population HRS is individuals aged 51 and older and their spouses.

For this study, data from waves 1–15 collected between 1992 and 2020 were used. Taking advantage of the HRS cohort sampling plan, we identified three birth cohorts: the HRS cohort, the War Babies (WBs), and the Early Baby Boomers (EBBs). The initial HRS cohort consists of individuals born between 1931 and 1941 who were aged 51–61 in 1992. The WBs were added in 1998 and include individuals born between 1942 and 1947, while the EBBs were added in 2004 and contain individuals born between 1948 and 1953. Individuals from the HRS cohort born between 1931 and 1935 (inclusive) were excluded from the sample because they entered the survey at ages 56 and older, and the initial part of their work histories is thus missing. Spouses of participants under 51 years of age were also excluded until they reached age 51. Our study sample included individuals who responded to at least two waves and had valid information on gender and education.

### Assessment of health and work states

**Figure 1.**
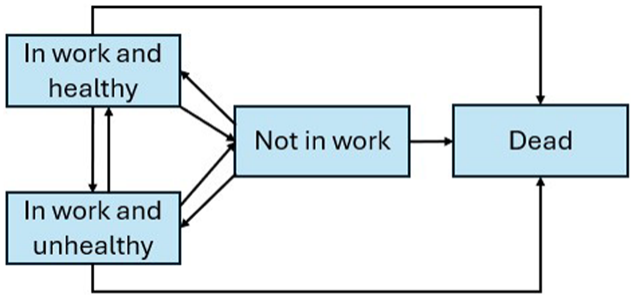
Multistate model of working LE. Permitted transitions are shown with arrows.

For each wave, we classified participants as dead or into one of three alive states (healthy and in work, unhealthy and in work, not in work). We defined an age-dependent transition model where transitions to any state can occur from any of the three alive states while the dead state acts as an absorbing state (Figure 1). We defined employment status by self-reported participation in paid employment obtained from the question “Are you currently working for pay?” We defined health status by the self-reported presence of health limitations affecting paid work derived from the question “Do you have any limitations or health problems that restrict the type or amount of paid work you can do?” This measure of health was used in previous studies estimating HWLE (Hambisa et al., 2023; Laaksonen et al., 2022) and was established as a valid measure to monitor trends in employment with disability (Burkhauser et al., 2002). For these two variables, valid responses were transformed into a dichotomous value of 1 or 0 (yes/no). We included the date of death via the Exit Interview conducted with a participant’s family member. We dichotomized both gender (men or women) and educational level (low or high). We consider only two educational groups to ensure model convergence: low education included individuals with a high school diploma or less, and high education included individuals with any post-secondary education.

### Statistical analysis

For this study, we estimated state-specific and marginal life expectancies with continuous-time multi-state models using *elect*, an add-on to the *msm* (multi-state modelling) package in R (Jackson, 2011; R Core Team, 2013; van den Hout et al., 2019). For those estimations, we estimated transition probabilities and a state distribution model using observed age-specific transitions between states in the longitudinal data. First, a general age-dependent multistate model following a Gompertz function was fitted with *msm* and used to derive transition probabilities from any of the three alive states to any states. Model parameters are estimated by maximum likelihood where observation times are interval censored and death times are exact, meaning transitions among the three alive states can take place anytime between two waves. Second, the age-specific state distribution is modelled using a multinomial regression model. Point estimates of life expectancies are then derived from the parameters in the multistate model and multinomial regression model, using numerical integration of the transition probabilities. We used the default “step” method for the numerical integration. Third, standard errors and 95% confidence intervals (CI) were estimated through a simulation-based method that computed life expectancies of repeated random draws. This method assumes that the model parameters follow a multivariate normal distribution, centered on their estimated values, with variability determined by the model’s covariance matrix. Multiple sets of parameter values are then randomly drawn from this distribution, and life expectancies are recalculated for each draw. We used 500 simulations for all 95% CI.

In this study, we estimated temporary expectancies for the states healthy and in work (HWLE), not healthy and in work (UHWLE), and not in work (NWLE). All expectancies consider ages 51–80. This means that HWLE is defined as the cumulative average number of years’ people are expected to be both healthy and in work between the ages of 51 and 80 years. Working life expectancy was calculated by summing values for the two working states (healthy and not healthy). Total life expectancy between ages 51 and 80 was calculated by summing expectancies for all three living states. Four age-dependent multistate models were fitted separately for each cohort with either no covariate (1), gender as a unique covariate (2), education as a unique covariate (3), or both gender and education as covariates (4).

### Sensitivity analyses

To assess the robustness of the cohort trends, we conducted four sets of sensitivity analyses. First, we used an alternative health definition, the self-report of health. Self-reported health was considered good if respondents reported excellent, very good, or good health and considered poor if respondents reported fair or poor health. Second, we used an alternative definition of employment, where anyone was not considered as working if they reported working less than 15 hr per week. Third, we used the age ranges of 51–70 years and 51–101 years to compute working and not working life expectancies. Fourth, we reran our analyses using only White/Caucasian individuals to ensure that the results are not driven by changes in ethnic composition between cohorts. Finally, since the life expectancies are estimated based on numerical approximation, we ran further sensitivity analyses using the alternative “MiddleRiemann” and “Simpson” methods for the numerical approximation, instead of the default “step” method.

## Results

The study sample comprised 13,969 individuals among the three cohorts (5,865 in HRS, 3,498 in WB, and 4,606 in EBB) for a total of 124,495 observations (see online Supplementary material, Supplementary Figure 1 for the flowchart of participant selection). The number of observations by individual ranged between 2 and 15, with an average of about 9. Characteristics for each cohort are shown in Table 1.

**Table 1.** Sample characteristics.

Cohort	Number of individuals	Number of records	Average number of records	Person-years
HRS	5,865	60,749	10.36	109,768
WB	3,498	32,443	9.27	57,890
EBB	4,606	31,303	6.80	53,394
Total	13,969	124,495	8.91	221,052

*Note*. HRS = Health and Retirement Study; WB = war baby; EBB = early baby boomer.

According to our estimates shown in Table 2, life expectancy (LE) between ages 51 and 80 has increased between cohorts, from 23.47 years (HRS cohort; 95% CI: 23.27–23.65) to 24.26 years (EBBs; 95% CI: 23.88–24.57). Working life expectancy (WLE) stayed roughly constant across cohorts, going from 10.03 years (95% CI: 9.84–10.21) among the HRS cohort to 10.21 years (95% CI: 9.96–10.42) among the EBBs. HWLE at age 51 was 8.95 years for the HRS cohort (95% CI: 8.79–9.13), 9.00 years for the WBs (95% CI: 8.77–9.21), and 9.08 years for the EBBs (95% CI: 8.86–9.30). UHWLE was 1.08 years for the HRS cohort (95% CI: 1.03–1.13), 1.13 years for the WBs (95% CI: 1.06–1.22), and 1.12 years for the EBBs (95% CI: 1.04–1.20).

**Table 2.** Life expectancies and working life expectancies between ages 51 and 80 in the United States by cohort, gender, and level of education.

Variables	Cohort	In work and healthy	In work and unhealthy	In work	Not in work	Life expectancy
**Total**	HRS	8.95	1.08	10.03	13.44	23.47
		(8.79-9.13)	(1.03-1.13)	(9.84-10.21)	(13.22-13.65)	(23.27-23.65)
	WB	9.00	1.13	10.13	14.11	24.23
		(8.77-9.21)	(1.06-1.22)	(9.90-10.34)	(13.82-14.36)	(23.95-24.45)
	EBB	9.08	1.12	10.21	14.05	24.26
		(8.86-9.30)	(1.04-1.20)	(9.96-10.42)	(13.64-14.44)	(23.88-24.57)
**Gender**						
**Men**	HRS	9.85	1.19	11.03	11.59	22.63
		(9.59-10.10)	(1.11-1.27)	(10.78-11.29)	(11.29-11.87)	(22.29-22.91)
	WB	10.21	1.21	11.42	11.99	23.4.0
		(9.82-10.57)	(1.09-1.33)	(11.02-11.78)	(11.51-12.38)	(22.92-23.81)
	EBB	9.79	1.09	10.88	12.57	23.45
		(9.45-10.09)	(0.98-1.21)	(10.52-11.20)	(12.05-13.01)	(22.90-23.87)
**Women**	HRS	8.17	1.00	9.17	15.06	24.23
		(7.94-8.37)	(0.93-1.08)	(8.92-9.38)	(14.75-15.32)	(23.93-24.46)
	WB	8.27	1.08	9.35	15.44	24.79
		(7.99-8.53)	(0.98-1.17)	(9.05-9.60)	(15.05-15.77)	(24.38-25.06)
	EBB	8.54	1.15	9.69	15.21	24.91
		(8.26-8.79)	(1.04-1.26)	(9.39-9.93)	(14.74-15.60)	(24.39-25.27)
**Education**						
**Low**	HRS	7.97	1.03	9.00	13.85	22.85
		(7.75-8.17)	(0.97-1.10)	(8.78-9.21)	(13.56-14.12)	(22.57-23.11)
	WB	7.76	1.01	8.77	14.71	23.48
		(7.46-8.01)	(0.91-1.10)	(8.45-9.05)	(14.24-15.08)	(22.97-23.81)
	EBB	7.38	0.96	8.34	14.90	23.25
		(7.10-7.64)	(0.87-1.07)	(8.05-8.63)	(14.36-15.34)	(22.68-23.68)
**High**	HRS	10.57	1.18	11.76	12.78	24.53
		(10.29-10.86)	(1.09-1.28)	(11.45-12.06)	(12.41-13.07)	(24.18-24.81)
	WB	10.39	1.27	11.66	13.45	25.11
		(10.06-10.72)	(1.14-1.39)	(11.30-12.00)	(12.98-13.78)	(24.69-25.42)
	EBB	10.47	1.27	11.74	13.36	25.09
		(10.16-10.74)	(1.15-1.39)	(11.40-12.02)	(12.89-13.74)	(24.63-25.41)
**Gender x Education**						
**Men (low)**	HRS	8.83	1.14	9.97	11.88	21.84
		(8.55-9.11)	(1.05-1.23)	(9.66-10.26)	(11.51-12.18)	(21.43-22.13)
	WB	8.93	1.11	10.04	12.49	22.54
		(8.52-9.31)	(0.97-1.23)	(9.57-10.45)	(11.83-12.96)	(21.77-22.94)
	EBB	8.01	0.93	8.93	13.23	22.16
		(7.65-8.35)	(0.81-1.04)	(8.55-9.30)	(12.50-13.77)	(21.35-22.68)
**Women (low)**	HRS	7.32	0.95	8.27	15.43	23.7
		(10.94-11.66)	(0.87-1.03)	(8.01-8.53)	(15.07-15.71)	(23.35-23.95)
	WB	7.15	0.95	8.10	15.97	24.07
		(10.92-11.86)	(0.83-1.05)	(7.77-8.40)	(15.47-16.35)	(23.55-24.41)
	EBB	6.98	0.98	7.96	16.07	24.03
		(10.59-11.42)	(0.86-1.10)	(7.66-8.25)	(15.42-16.56)	(23.40-24.49)
**Men (high)**	HRS	11.33	1.27	12.60	11.21	23.8
		(7.07-7.57)	(1.15-1.39)	(12.18-12.93)	(10.80-11.56)	(21.35-22.68)
	WB	11.43	1.31	12.74	11.56	24.30
		(6.85-7.45)	(1.15-1.48)	(12.17-13.16)	(10.99-12.01)	(23.68-24.71)
	EBB	11.04	1.22	12.26	12.13	24.39
		(6.68-7.29)	(1.07-1.38)	(11.75-12.66)	(11.52-12.59)	(23.77-24.81)
**Women (high)**	HRS	9.79	1.1	10.89	14.37	25.25
		(9.45-10.10)	(0.99-1.20)	(10.51-11.24)	(13.91-14.75)	(24.86-25.53)
	WB	9.67	1.24	10.90	14.80	25.71
		(9.28-10.02)	(1.09-1.38)	(10.47-11.29)	(14.25-15.23)	(25.19-26.01)
	EBB	9.98	1.33	11.31	14.41	25.71
		(9.61-10.32)	(1.17-1.48)	(10.91-11.67)	(13.81-14.83)	(25.12-26.08)

*Note*. HRS = Health and Retirement Study; WB = war baby; EBB = early baby boomer. Values represent estimates with confidence intervals in parentheses.

Significant differences in LE and working life expectancy were observed between men and women. First, LE between ages 51 and 80 was higher for women in all three cohorts, with an ∼1.5-year advantage over men. Although not significant, both groups saw increases in LE between the HRS cohort and the EBBs. For men, gains in LE were made not working, from 11.59 years (95% CI: 11.29–11.87) to 12.57 years (95% CI: 12.05–13.01). Still, years not in work were greater for women compared to men for all three cohorts. For women, WLE increased between the HRS cohort and the EBBs, from 9.17 years (95% CI: 8.92–9.38) to 9.69 years (95% CI: 9.39–9.93), but a larger portion of working life was spent unheal­thily. Second, on average, men had higher WLE and HWLE than women, but the gap narrowed between cohorts. UHWLE was also significantly higher for men (1.19 years; 95% CI: 1.11–1.27) than for women (1.00 years; 95% CI: 0.93–1.08) in the HRS cohort, but no significant difference could be detected for the WBs and EBBs.

Estimates of working life expectancy showed important disparities across educational groups. Among all three cohorts, there was a large advantage in WLE for more highly educated individuals. That advantage also increased over time due to a significant decrease in WLE among the lower educational group from 9.00 years (HRS; 95% CI: 8.78–9.21) to 8.34 years (EBB; 95% CI: 8.05–8.63). Similarly, significant differences in HWLE were observed between educational groups. While HWLE remained stable among more highly educated individuals (around 10.5 years), a significant decrease in HWLE was observed among their less highly educated counterparts, going from 7.97 years (95% CI: 7.75–8.17) to 7.38 years (95% CI: 7.10–7.64), thus widening the gap between them. Among the WBs and EBBs, estimates of UHWLE also highlighted significant differences between these groups, with more years spent working unhealthily among the more highly educated. There was no significant change in UHWLE across cohorts regardless of educational attainment.

When gender and education were combined, estimations of HWLE indicated that highly educated men had the highest HWLE while less highly educated women had the lowest. This difference persisted across cohorts. HWLE decreased between the HRS and EBB cohorts for less educated men, from 8.83 years (95% CI: 8.55–9.11) to 8.01 years (95% CI: 7.65–8.35). Less educated men also saw their WLE decrease from 9.97 years (95% CI: 9.66–10.26) to 8.93 years (95% CI: 8.55–9.30). Consequently, these decreases in HWLE and WLE across cohorts increased the disparities in working years between less educated men and more educated men. Similarly, the gap in HWLE and UHWLE between less highly and more highly educated women increased across cohorts. Overall, gains in HWLE across cohorts, even if in most cases insignificant, were always accompanied by gains in UHWLE.

### Sensitivity analyses

When using an alternative definition to measure health, the prevalence of poor health was more important than with the self-assessed limitations of work activities, which translated into a lower HWLE and a greater UHWLE. However, the trends across birth cohorts and disparities between genders and educational levels remained consistent with our main findings (see online Supplementary material, Supplementary Table 1). Similarly, restraining the definition of employment to at least 15 hr of work per week also did not result in any changes in the trends across cohorts and disparities between subpopulation groups (see online Supplementary material, Supplementary Table 2). When using alternative age ranges, the pattern of change between cohorts (in terms of the different expectancies) was roughly the same for both age ranges of 51–70 and 51–101 years (see online Supplementary material, Supplementary Tables 3 and 4). HWLE values across cohorts were similar to those in the main analysis when considering the White/Caucasian population only. This suggests that the increase in the share of non-White/Caucasian among the younger cohorts did not significantly impact the comparison of HWLE across cohorts (see online Supplementary material, Supplementary Table 5). Life expectancy estimates based on alternative methods for numerical approximation were very similar, indicating that the results are insensitive to the numerical approximation method (see online Supplementary material, Supplementary Tables 6 and 7).

## Discussion

This study found that the expected number of years spent healthy and working was significantly lower than the full-­pension age among all cohorts examined. In addition, although some increases in HWLE were found across cohorts, these were insignificant and would otherwise be insufficient to compensate for the 1-year increase in the full retirement age that affected them. Furthermore, where there were significant differences across cohorts, they indicated either a deterioration of HWLE or an increase in the number of years spent unhealthy and working. For younger cohorts approaching retirement, these trends could suggest either longer working lives in poor health or reduced Social Security benefits.

The lack of improvement in WLE and HWLE in the entire population masked disparities in the trends between genders and groups of educational levels. In fact, only younger cohorts of women could expect to work longer between the ages of 51 and 80 than the cohorts that preceded them. However, women’s gains in WLE were mostly driven by an increasing labor force participation among younger cohorts, rather than by an actual extension of working life. In fact, supplementary analyses found that the working LE of women already working at age 51 barely increased across cohorts, meaning that gains in working life expectancy among them are mainly due to increases in labor force participation at subsequent ages (see online Supplementary material, Supplementary Tables 8 and 9). In line with other studies, we found important educational disparities where less highly educated individuals had a much lower HWLE between ages 51 and 80 than their more highly educated counterparts (Hambisa et al., 2023; Parker et al., 2020; Sperlich et al., 2023). Moreover, less highly educated individuals in younger cohorts had a significantly lower HWLE than those in older cohorts. Our study is thus the first to report lower HWLE among younger cohorts within a given educational group. This highlights the importance of developing policies that address not only the disparities between population subgroups but also how these evolve across cohorts. Overall, these trends suggest a deterioration over time in the ability of Americans to work at older ages.

Compared to the HRS cohort, the WBs and EBBs had, on average, greater incentives to retire later, as they were affected by a higher normal retirement age, reduced early retirement benefits, and a greater number of them had defined contribution pension plans. Despite this, among every EBB population subgroup, only more educated female EBBs had a greater working life expectancy between ages 51 and 80 compared to their counterparts in the HRS cohort. Yet, more than half of this difference was attributable to gains in UHWLE. In other words, gains in WLE across cohorts were associated with gains in UHWLE, while losses in WLE were associated with losses in HWLE. Thus, gains in WLE, if there were any, were mostly made thanks to less healthy workers staying longer on the labor market.

Meanwhile, the lack of increase in WLE may reflect the incapacity of workers to remain employed as they age, despite their desire to continue working. This stagnation in WLE had previously been reported for the United States (Dudel & Myrskylä, 2020), and can, on the one hand, this may be due to the tightening of the labor market following the 2008 financial crisis. On the other hand, our results also suggest that poor health could be at play. As the younger cohorts born between 1954 and 1960 are now seeing their retirement age increase from 66 to 67 years and facing increased early retirement penalties, research is sorely needed to determine the exact extent to which poor health forms an impediment to longer working lives among Americans.

To address the concerns that population aging raises about the sustainability of the pension system, policies that aim to extend the HWLE of future cohorts of older Americans will be crucial. In order to achieve longer and healthier working lives, particular attention should be placed on two key elements: increasing labor force participation among healthy working-­age individuals and improving work capacity at older ages. First, pension policies that focus on extending working lives only affect those already working. However, only about half of Americans are steadily employed in their 50s (Berkman & Truesdale, 2023). Furthermore, policies that address workers’ needs before the normal pension age seem essential. Safer, empowering, and more flexible and inclusive work environments could encourage Americans to work more at older ages (Hall & Richter, 1988; Sinclair et al., 2024; Tremblay & Genin, 2009). Second, as our study has shown, policies that promote longer economically active lives in a context of declining health often led to reduced retirement benefits, widened disparities, and added years spent working in poor health. Therefore, the need for policies that foster healthier workplaces for older workers, especially for those more at risk of working in poor health, is urgent. For example, the promotion of healthy lifestyles, accommodations for workers with health conditions, or targeted interventions could help older Americans extend their working lives (Crawford et al., 2010; Hansson et al., 1997; Leijten et al., 2013).

Previous studies on HWLE used different measures to assess health statuses, such as the presence of limiting long-standing illnesses or chronic diseases. Using the presence of chronic diseases as a health measure allows researchers to identify which diseases are associated with a higher risk of working with poor health and propose more targeted policies. These health measures are also less subjective and entertain a less endogenous relationship with the decision to work. However, they do not necessarily capture an individual’s true capacity to work, as they do not capture their health in relation to the mental and physical demands of their job. Hence, the strength of the health measure used in this study, the presence of health problems limiting work activities, is that it is more sensitive to different work contexts.

This study also contains certain limitations. First, our models did not consider transitions among non-working states, as other studies did. Estimating trends in healthy and unhealthy non-working life expectancies across cohorts could help identify where potential gains in HWLE could be made. However, rarely observed transitions between some states hindered model convergence and estimation of confidence intervals for some subgroups, making it impossible to establish a five-state model as in other studies.

Second, our longitudinal design meant that the maximum follow-up age was lower in each subsequent cohort, where the WBs were followed until ages 73–78 and the EBBs until ages 67–72 (whereas most of the HRS cohort members were followed until age 80). As a result, we needed to resort to an extrapolation of the observed transition rates to obtain expectancies for the full 51–80 years age range. However, based on the observed transitions among the HRS cohort, the extrapolation among the WB and EBB concerned time that would otherwise mostly have been spent not working. Therefore, we consider it unlikely that these lower maximum follow-up ages have affected the comparison of the time spent working across cohorts.

Third, several characteristics that can influence the length of healthy working lives were not taken into consideration. These include, for example, a person’s income, their type of occupation, or the extent to which they have a healthy lifestyle. A study conducted in China reported important disparities in HWLE between population subgroups using these characteristics (Li et al., 2024). For example, the time spent working unhealthy was reported to be longer for the rural population, agricultural laborers, and people with low education, whereas urban population, enterprise employees, and people with high education or income had more-healthy non-working years. Thus, future research in the United States that considers these characteristics could help policymakers target sub-populations more at risk of working in poor health. Additionally, racial disparities in access to healthcare, education, and employment in the United States are largely documented (Brittain & Kozlak, 2007; Doede, 2016; Yearby, 2018). Minority and ethnic groups accounted for a larger proportion of the size of each younger cohort (see online Supplementary material, Supplementary Table 10), and this change could account for some of the changes in HWLE and WLE found in this study. Monitoring trends in HWLE between ethnic groups should be an important consideration for future research.

This study was the first to monitor the health and work of individuals from a given age onward and compare those born in different time periods in terms of HWLE. Our findings showed that HWLE failed to increase between birth cohorts for all population subgroups. This heavily contrasts with the rising self-reported probability of working at older ages among the same cohorts of Americans (Dong et al., 2017). With the present deterioration of the health status among the new generations of working-age individuals, it seems increasingly unlikely that many will be able to extend their working lives. As there is an increased pressure on American workers to postpone retirement, policies that promote longer and healthier working lives are going to be crucial for alleviating the disadvantages workers face in front of retirement.

## Supplementary Material

gbaf119_Supplementary_Data

## Data Availability

HRS data can be accessed via the HRS Data Portal: https://hrsdata.isr.umich.edu/ All statistical models for the calculation of HWLE are based on R software version 4.4.2. Additional custom codes that support the findings of this study are available at https://doi.org/10.5281/zenodo.14290095
