# Correction to: Healthy working life expectancy across birth cohorts in the United States

**DOI:** 10.1093/geronb/gbaf153

**Published:** 2025-10-07

This is a correction to: Blain, F., & Boissonneault, M. (2025). Healthy working life expectancy across birth cohorts in the United States. *The Journals of Gerontology, Series B: Psychological Sciences and Social Sciences*, 80(8), gbaf119. https://doi.org/10.1093/geronb/gbaf119

In the original version of the article, Table 2 contained an earlier version of the table submitted for review for publication consideration. The correct Table 2 and heading should read as “**Table 2.** Life expectancies and working life expectancies between ages 51 and 80 in the United States by cohort, gender, and level of education” instead of originally-published **Table 2.** Life expectancies and working life expectancies at age 51 in the United States.

This emendation has been made within the article.

**Table 2.** Life expectancies and working life expectancies at age 51 in the United States.

Variables	Cohort	Life expectancy	In work and healthy	In work and unhealthy	Not in work	In work
**Total**	HRS	29.14	9.39	1.14	18.61	10.53
		(28.63, 29.61)	(9.22, 9.58)	(1.08, 1.20)	(18.11, 19.12)	(10.32, 10.72)
	WB	31.5	9.41	1.22	20.87	10.64
		(30.15, 32.71)	(9.15, 9.62)	(1.14, 1.33)	(19.58, 22.02)	(10.38, 10.87)
	EBB	32.77	9.5	1.21	22.06	10.71
		(30.41, 34.77)	(9.27, 9.73)	(1.10, 1.33)	(19.68, 24.09)	(10.44, 10.96)
**Gender**						
**Men**	HRS	27.14	10.33	1.26	15.56	11.59
		(26.51, 27.68)	(10.05, 10.60)	(1.17, 1.35)	(14.98, 16.07)	(11.31, 11.85)
	WB	29.26	10.65	1.30	17.31	11.95
		(27.85, 30.44)	(10.22, 11.02)	(1.17, 1.45)	(16.08, 18.52)	(11.51, 12.33)
	EBB	30.42	10.21	1.17	19.04	11.38
		(28.19, 32.55)	(9.84, 10.52)	(1.03, 1.32)	(16.84, 21.12)	(10.98, 11.74)
**Women**	HRS	31.18	8.56	1.05	21.56	9.62
		(30.37, 31.83)	(8.31, 8.78)	(0.97, 1.14)	(20.78, 22.18)	(9.35, 9.84)
	WB	33.46	8.67	1.16	23.63	9.83
		(31.84, 34.76)	(8.36, 8.94)	(1.05, 1.28)	(22.05, 24.92)	(9.49, 10.08)
	EBB	34.91	8.95	1.24	24.71	10.19
		(32.22, 36.99)	(8.65, 9.20)	(1.10, 1.40)	(22.10, 26.82)	(9.86, 10.47)
**Education**						
**Low**	HRS	27.72	8.38	1.07	18.27	9.45
		(27.13, 28.25)	(8.14, 8.59)	(1.01, 1.14)	(17.71, 18.74)	(9.21, 9.67)
	WB	29.64	8.12	1.07	20.45	9.19
		(28.24, 30.89)	(7.81, 8.39)	(0.97, 1.19)	(19.12, 21.65)	(8.86, 9.49)
	EBB	30.46	7.76	1.02	21.68	8.78
		(27.96, 32.64)	(7.45, 8.01)	(0.91, 1.16)	(19.28, 23.86)	(8.46, 9.07)
**High**	HRS	31.60	11.11	1.26	19.23	12.37
		(30.77, 32.28)	(10.80, 11.41)	(1.16, 1.37)	(18.44, 19.90)	(12.03, 12.70)
	WB	33.77	10.87	1.40	21.50	12.27
		(32.33, 35.06)	(10.52, 11.21)	(1.26, 1.54)	(20.10, 22.78)	(11.86, 12.63)
	EBB	34.70	10.93	1.38	22.39	12.31
		(32.21, 26.80)	(10.60, 11.22)	(1.24, 1.57)	(20.06, 24.44)	(11.93, 12.65)
**Gender × Education**						
**Men (low)**	HRS	25.36	9.22	1.18	14.96	10.4
		(24.63, 25.91)	(8.91, 9.51)	(1.09, 1.27)	(14.34, 15.49)	(10.07, 10.72)
	WB	27.29	9.31	1.18	16.80	10.49
		(25.68, 28.61)	(8.87, 9.70)	(1.02, 1.31)	(15.33, 18.08)	(9.98, 10.91)
	EBB	27.73	8.4	0.98	18.36	9.38
		(25.10, 30.00)	(8.00, 8.75)	(0.84, 1.11)	(15.87, 20.56)	(8.92, 9.75)
**Women (low)**	HRS	29.65	11.93	1.35	16.36	13.28
		(28.71, 30.42)	(11,50, 12.29)	(1.23, 1.49)	(15.53, 17.08)	(12.80, 13.65)
	WB	31.47	11.94	1.44	18.09	13.38
		(29.69, 32.82)	(11.36, 12.39)	(1.25, 1.65)	(16.47, 19.38)	(12.70, 13.85)
	EBB	32.46	11.5	1.32	19.65	12.81
		(29.65, 34.74)	(11.00, 11.87)	(1.13, 1.54)	(16.99, 21.90)	(12.23, 13.65)
**Men (high)**	HRS	29.87	7.69	1.00	21.18	8.69
		(29.05, 30.54)	(7.43, 7.96)	(0.91, 1.08)	(20.36, 21.84)	(8.41, 8.95)
	WB	31.54	7.49	1.01	23.04	8.50
		(29.81, 32.93)	(7.17, 7.81)	(0.88, 1.12)	(21.35, 24.36)	(8.16, 8.81)
	EBB	32.72	7.36	1.04	24.33	8.40
		(29.84, 34.96)	(7.04, 7.67)	(0.91, 1.18)	(21.43, 26.48)	(8.06, 8.71)
**Women (high)**	HRS	33.88	10.28	1.17	22.44	11.45
		(32.80, 34.75)	(9.90, 10.61)	(1.05, 1.29)	(21.38, 23.25)	(11.02, 11.84)
	WB	35.97	10.13	1.35	24.49	11.48
		(33.99, 37.47)	(9.71, 10.49)	(1.18, 1.53)	(22.59, 25.97)	(10.99, 11.88)
	EBB	36.94	10.43	1.45	25.07	11.87
		(34.03, 39.08)	(10.04, 10.78)	(1.25, 1.65)	(22.30, 27.13)	(11.40, 12.26)

*Note*. HRS = Health and Retirement Study; WB = war baby; EBB = early baby boomer. Values represent estimates with confidence intervals in parentheses.

**Table 2.** Life expectancies and working life expectancies between ages 51 and 80 in the United States by cohort, gender, and level of education.

Variables	Cohort	In work and healthy	In work and unhealthy	In work	Not in work	Life expectancy
**Total**	HRS	8.95	1.08	10.03	13.44	23.47
		(8.79-9.13)	(1.03-1.13)	(9.84-10.21)	(13.22-13.65)	(23.27-23.65)
	WB	9.00	1.13	10.13	14.11	24.23
		(8.77-9.21)	(1.06-1.22)	(9.90-10.34)	(13.82-14.36)	(23.95-24.45)
	EBB	9.08	1.12	10.21	14.05	24.26
		(8.86-9.30)	(1.04-1.20)	(9.96-10.42)	(13.64-14.44)	(23.88-24.57)
**Gender**						
**Men**	HRS	9.85	1.19	11.03	11.59	22.63
		(9.59-10.10)	(1.11-1.27)	(10.78-11.29)	(11.29-11.87)	(22.29-22.91)
	WB	10.21	1.21	11.42	11.99	23.4.0
		(9.82-10.57)	(1.09-1.33)	(11.02-11.78)	(11.51-12.38)	(22.92-23.81)
	EBB	9.79	1.09	10.88	12.57	23.45
		(9.45-10.09)	(0.98-1.21)	(10.52-11.20)	(12.05-13.01)	(22.90-23.87)
**Women**	HRS	8.17	1.00	9.17	15.06	24.23
		(7.94-8.37)	(0.93-1.08)	(8.92-9.38)	(14.75-15.32)	(23.93-24.46)
	WB	8.27	1.08	9.35	15.44	24.79
		(7.99-8.53)	(0.98-1.17)	(9.05-9.60)	(15.05-15.77)	(24.38-25.06)
	EBB	8.54	1.15	9.69	15.21	24.91
		(8.26-8.79)	(1.04-1.26)	(9.39-9.93)	(14.74-15.60)	(24.39-25.27)
**Education**						
**Low**	HRS	7.97	1.03	9.00	13.85	22.85
		(7.75-8.17)	(0.97-1.10)	(8.78-9.21)	(13.56-14.12)	(22.57-23.11)
	WB	7.76	1.01	8.77	14.71	23.48
		(7.46-8.01)	(0.91-1.10)	(8.45-9.05)	(14.24-15.08)	(22.97-23.81)
	EBB	7.38	0.96	8.34	14.90	23.25
		(7.10-7.64)	(0.87-1.07)	(8.05-8.63)	(14.36-15.34)	(22.68-23.68)
**High**	HRS	10.57	1.18	11.76	12.78	24.53
		(10.29-10.86)	(1.09-1.28)	(11.45-12.06)	(12.41-13.07)	(24.18-24.81)
	WB	10.39	1.27	11.66	13.45	25.11
		(10.06-10.72)	(1.14-1.39)	(11.30-12.00)	(12.98-13.78)	(24.69-25.42)
	EBB	10.47	1.27	11.74	13.36	25.09
		(10.16-10.74)	(1.15-1.39)	(11.40-12.02)	(12.89-13.74)	(24.63-25.41)
**Gender x Education**						
**Men (low)**	HRS	8.83	1.14	9.97	11.88	21.84
		(8.55-9.11)	(1.05-1.23)	(9.66-10.26)	(11.51-12.18)	(21.43-22.13)
	WB	8.93	1.11	10.04	12.49	22.54
		(8.52-9.31)	(0.97-1.23)	(9.57-10.45)	(11.83-12.96)	(21.77-22.94)
	EBB	8.01	0.93	8.93	13.23	22.16
		(7.65-8.35)	(0.81-1.04)	(8.55-9.30)	(12.50-13.77)	(21.35-22.68)
**Women (low)**	HRS	7.32	0.95	8.27	15.43	23.7
		(10.94-11.66)	(0.87-1.03)	(8.01-8.53)	(15.07-15.71)	(23.35-23.95)
	WB	7.15	0.95	8.10	15.97	24.07
		(10.92-11.86)	(0.83-1.05)	(7.77-8.40)	(15.47-16.35)	(23.55-24.41)
	EBB	6.98	0.98	7.96	16.07	24.03
		(10.59-11.42)	(0.86-1.10)	(7.66-8.25)	(15.42-16.56)	(23.40-24.49)
**Men (high)**	HRS	11.33	1.27	12.60	11.21	23.8
		(7.07-7.57)	(1.15-1.39)	(12.18-12.93)	(10.80-11.56)	(21.35-22.68)
	WB	11.43	1.31	12.74	11.56	24.30
		(6.85-7.45)	(1.15-1.48)	(12.17-13.16)	(10.99-12.01)	(23.68-24.71)
	EBB	11.04	1.22	12.26	12.13	24.39
		(6.68-7.29)	(1.07-1.38)	(11.75-12.66)	(11.52-12.59)	(23.77-24.81)
**Women (high)**	HRS	9.79	1.1	10.89	14.37	25.25
		(9.45-10.10)	(0.99-1.20)	(10.51-11.24)	(13.91-14.75)	(24.86-25.53)
	WB	9.67	1.24	10.90	14.80	25.71
		(9.28-10.02)	(1.09-1.38)	(10.47-11.29)	(14.25-15.23)	(25.19-26.01)
	EBB	9.98	1.33	11.31	14.41	25.71
		(9.61-10.32)	(1.17-1.48)	(10.91-11.67)	(13.81-14.83)	(25.12-26.08)

*Note*. HRS = Health and Retirement Study; WB = war baby; EBB = early baby boomer. Values represent estimates with confidence intervals in parentheses.

