# Supplementary material for: Healthy working life expectancy across birth cohorts in the United States
Source: J Gerontol B Psychol Sci Soc Sci. 2025 Jun 27;80(8):gbaf119. doi: 10.1093/geronb/gbaf119 (PMC12313020; doi:10.1093/geronb/gbaf119)
Supplement: gbaf119_Supplementary_Data [file gbaf119_supplementary_data.docx]

***The Journals of Gerontology, Series B: Psychological Sciences and Social Sciences* Supplementary Material: Blain & Boissonneault. Healthy Working Life Expectancy across birth cohorts in the United States.**

Supplementary Figure 1. Flowchart of participant selection.

**
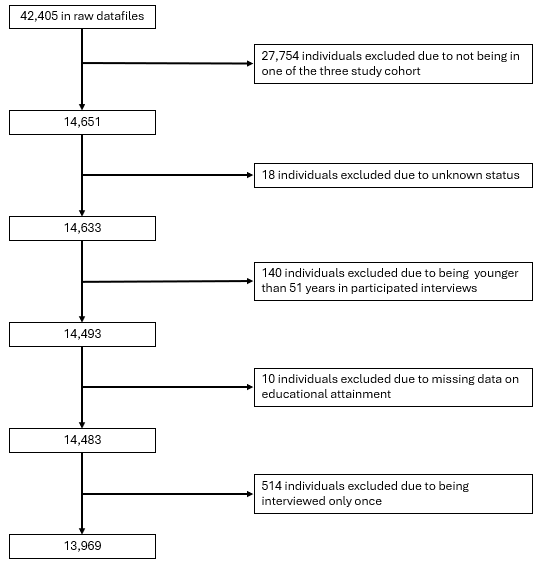
**

Supplementary Table 1. Sensitivity analysis of life expectancies between ages 51 and 80 years by cohort, gender, and level of education using self-rated health

| Variables | Cohort | Life expectancy | In work and healthy | In work and unhealthy | In work | Not in work |
| --- | --- | --- | --- | --- | --- | --- |
| Total | HRS | 23.45 | 8.61 | 1.45 | 10.06 | 13.4 |
|  |  | (23.25 to 23.64) | (8.45 to 8.79) | (1.38 to 1.51) | (9.87 to 10.23) | (13.17 to 13.60) |
|  | WB | 24.24 | 8.79 | 1.37 | 10.16 | 14.08 |
|  |  | (23.93 to 24.47) | (8.55 to 9.00) | (1.28 to 1.46) | (9.91 to 10.39) | (13.72 to 14.36) |
|  | EBB | 24.25 | 8.6 | 1.56 | 10.16 | 14.09 |
|  |  | (23.87 to 24.57) | (8.38 to 8.81) | (1.47 to 1.64) | (9.93 to 10.38) | (13.68 to 14.46) |
| Gender |  |  |  |  |  |  |
| Men | HRS | 22.61 | 9.42 | 1.64 | 11.05 | 11.56 |
|  |  | (22.29 to 22.92) | (9.15 to 9.68) | (1.54 to 1.74) | (10.79 to 11.31) | (11.25 to 11.83) |
|  | WB | 23.41 | 9.93 | 1.53 | 11.46 | 11.95 |
|  |  | (22.92 to 23.82) | (9.53 to 10.28) | (1.4 to 1.68) | (11.06 to 11.83) | (11.46 to 12.35) |
|  | EBB | 23.43 | 9.19 | 1.63 | 10.81 | 12.61 |
|  |  | (22.84 to 23.83) | (8.83 to 9.50) | (1.50 to 1.77) | (10.45 to 11.14) | (12.08 to 13.04) |
| Women | HRS | 24.23 | 7.92 | 1.29 | 9.21 | 15.02 |
|  |  | (23.94 to 24.46) | (7.70 to 8.13) | (1.20 to 1.38) | (8.96 to 9.42) | (14.72 to 15.29) |
|  | WB | 24.78 | 8.11 | 1.26 | 9.38 | 15.40 |
|  |  | (24.37 to 25.05) | (7.83 to 8.38) | (1.16 to 1.37) | (9.09 to 9.63) | (15 to 15.73) |
|  | EBB | 24.91 | 8.15 | 1.52 | 9.67 | 15.24 |
|  |  | (24.39 to 25.26) | (7.86 to 8.41) | (1.40 to 1.63) | (9.38 to 9.92) | (14.75 to 15.62) |
| Education |  |  |  |  |  |  |
| Low | HRS | 22.85 | 7.27 | 1.74 | 9.01 | 13.83 |
|  |  | (22.57 to 23.1) | (7.07 to 7.47) | (1.65 to 1.84) | (8.80 to 9.23) | (13.54 to 14.11) |
|  | WB | 23.49 | 7.16 | 1.64 | 8.80 | 14.69 |
|  |  | (23.01 to 23.82) | (6.88 to 7.42) | (1.51 to 1.76) | (8.48 to 9.09) | (14.23 to 15.07) |
|  | EBB | 23.23 | 6.20 | 2.10 | 8.30 | 14.93 |
|  |  | (22.65 to 23.66) | (5.94 to 6.45) | (1.95 to 2.25) | (8.00 to 8.58) | (14.40 to 15.39) |
| High | HRS | 24,54 | 10,81 | 1.00 | 11,81 | 12,73 |
|  |  | (24.21 to 24.82) | (10.51 to 11.09) | (0.91 to 1.10) | (11.49 to 12.12) | (12.37 to 13.03) |
|  | WB | 25,11 | 10,61 | 1,08 | 11,69 | 13,41 |
|  |  | (24.69 to 25.43) | (10.27 to 10.94) | (0.97 to 1.20) | (11.33 to 12.03) | (12.95 to 13.75) |
|  | EBB | 25,09 | 10,52 | 1,15 | 11,68 | 13,42 |
|  |  | (24.63 to 25.42) | (10.21 to 10.80) | (1.05 to 1.26) | (11.34 to 11.97) | (12.94 to 13.80) |
| Gender x Education |  |  |  |  |  |  |
| Men (low) | HRS | 21,84 | 7,96 | 2,01 | 9,97 | 11,87 |
|  |  | (21.45 to 22.13) | (7.69 to 8.22) | (1.88 to 2.13) | (9.67 to 10.25) | (11.52 to 12.17) |
|  | WB | 22,57 | 8,19 | 1,93 | 10,12 | 12,45 |
|  |  | (21.83 to 22.97) | (7.78 to 8.56) | (1.73 to 2.10) | (9.66 to 10.52) | (11.79 to 12.90) |
|  | EBB | 22,11 | 6,56 | 2,26 | 8,82 | 13,30 |
|  |  | (21.31 to 22.65) | (6.24 to 6.90) | (2.05 to 2.44) | (8.42 to 9.20) | (12.59 to 13.88) |
| Women (low) | HRS | 23,68 | 6,76 | 1,51 | 8,27 | 15,41 |
|  |  | (23.30 to 24.11) | (6.52 to 7.00) | (1.39 to 1.61) | (8.02 to 8.52) | (15.05 to 15.69) |
|  | WB | 24,06 | 6,62 | 1,48 | 8,10 | 15,96 |
|  |  | (23.68 to 24.72) | (6.34 to 6.92) | (1.33 to 1.62) | (7.78 to 8.40) | (15.44 to 16.34) |
|  | EBB | 24,03 | 5,97 | 1,99 | 7,96 | 16,07 |
|  |  | (23.75 to 24.81) | (5.69 to 6.25) | (1.81 to 2.16) | (7.63 to 8.27) | (15.43 to 16.58) |
| Men (high) | HRS | 23,75 | 11,49 | 1,13 | 12,61 | 11,14 |
|  |  | (23.34 to 23.93) | (11.10 to 11.82) | (1.02 to 1.24) | (12.21 to 12.95) | (10.73 to 11.49) |
|  | WB | 24,30 | 11,57 | 1,18 | 12,76 | 11,54 |
|  |  | (23.55 to 24.39) | (11.06 to 12.01) | (1.04 to 1.34) | (12.19 to 13.19) | (10.96 to 12.00) |
|  | EBB | 24,40 | 10,99 | 1,22 | 12,21 | 12,19 |
|  |  | (23.40 to 24.48) | (10.56 to 11.36) | (1.08 to 1.36) | (11.75 to 12.62) | (11.59 to 12.66) |
| Women (high) | HRS | 25,30 | 10,09 | 0,89 | 10,98 | 14,33 |
|  |  | (24.92 to 25.58) | (9.75 to 10.42) | (0.79 to 0.98) | (10.58 to 11.32) | (13.88 to 14.73) |
|  | WB | 25,70 | 9,93 | 1,01 | 10,94 | 14,76 |
|  |  | (25.20 to 26.02) | (9.54 to 10.29) | (0.89 to 1.14) | (10.52 to 11.32) | (14.23 to 15.19) |
|  | EBB | 25,69 | 10,11 | 1,1 | 11,21 | 14,48 |
|  |  | (25.11 to 26.05) | (9.75 to 10.48) | (0.98 to 1.23) | (10.83 to 11.55) | (13.89 to 14.91) |

Supplementary Table 2. Sensitivity analysis of life expectancies between ages 51 and 80 years by cohort, gender, and level of education using a 15 hours of work per week cutoff for employment status

| Variables | Cohort | Life expectancy | In work and healthy | In work and unhealthy | In work | Not in work |
| --- | --- | --- | --- | --- | --- | --- |
| Total | HRS | 23.48 | 8.32 | 0.93 | 9.26 | 14.22 |
|  |  | (23.28 to 23.66) | (8.18 to 8.50) | (0.88 to 0.98) | (9.08 to 9.43) | (14.00 to 14.43) |
|  | WB | 24.24 | 8.31 | 0.95 | 9.26 | 14.98 |
|  |  | (23.94 to 24.47) | (8.08 to 8.50) | (0.88 to 1.02) | (9.03 to 9.48) | (14.66 to 15.24) |
|  | EBB | 24.27 | 8.49 | 0.93 | 9.42 | 14.86 |
|  |  | (23.89 to 24.59) | (8.29 to 8.69) | (0.85 to 0.99) | (9.20 to 9.61) | (14.46 to 15.23) |
| Gender |  |  |  |  |  |  |
| Men | HRS | 22.65 | 9.28 | 1.05 | 10.32 | 12.32 |
|  |  | (22.30 to 22.93) | (9.03 to 9.52) | (0.97 to 1.12) | (10.09 to 10.57) | (12.02 to 12.60) |
|  | WB | 23.41 | 9.60 | 1.03 | 10.63 | 12.78 |
|  |  | (22.93 to 23.81) | (9.23 to 9.93) | (0.92 to 1.14) | (10.26 to 10.98) | (12.32 to 13.16) |
|  | EBB | 23.46 | 9.28 | 0.93 | 10.21 | 13.25 |
|  |  | (22.90 to 23.88) | (8.96 to 9.58) | (0.83 to 1.03) | (9.88 to 10.51) | (12.71 to 13.67) |
| Women | HRS | 24.22 | 7.48 | 0.84 | 8.32 | 15.9 |
|  |  | (23.92 to 24.45) | (7.27 to 7.68) | (0.77 to 0.91) | (8.10 to 8.52) | (15.59 to 16.16) |
|  | WB | 24.79 | 7.54 | 0.90 | 8.43 | 16.36 |
|  |  | (24.38 to 25.06) | (7.27 to 7.77) | (0.81 to 0.98) | (8.17 to 8.66) | (15.95 to 16.68) |
|  | EBB | 24.93 | 7.88 | 0.93 | 8.80 | 16.13 |
|  |  | (24.41 to 25.29) | (7.62 to 8.11) | (0.83 to 1.02) | (8.53 to 9.01) | (15.65 to 16.50) |
| Education |  |  |  |  |  |  |
| Low | HRS | 22.89 | 7.52 | 0.90 | 8.42 | 14.47 |
|  |  | (22.60 to 23.14) | (7.32 to 7.71) | (0.84 to 0.96) | (8.21 to 8.62) | (14.17 to 14.74) |
|  | WB | 23.50 | 7.32 | 0.87 | 8.19 | 15.31 |
|  |  | (22.98 to 23.82) | (7.05 to 7.57) | (0.79 to 0.96) | (7.89 to 8.46) | (14.83 to 15.67) |
|  | EBB | 23.28 | 7.02 | 0.81 | 7.83 | 15.45 |
|  |  | (22.70 to 23.71) | (6.74 to 7.26) | (0.73 to 0.91) | (7.56 to 8.10) | (14.90 to 15.88) |
| High | HRS | 24.51 | 9.67 | 1.01 | 10.67 | 13.84 |
|  |  | (24.16 to 24.80) | (9.40 to 9.93) | (0.92 to 1.10) | (10.38 to 10.96) | (13.48 to 14.14) |
|  | WB | 25.11 | 9.43 | 1.05 | 10.47 | 14.63 |
|  |  | (24.69 to 25.42) | (9.12 to 9.73) | (0.93 to 1.16) | (10.14 to 10.79) | (14.16 to 14.96) |
|  | EBB | 25.11 | 9.68 | 1.03 | 10.71 | 14.39 |
|  |  | (24.64 to 25.43) | (9.41 to 9.94) | (0.93 to 1.12) | (10.41 to 10.97) | (13.93 to 14.76) |
| Gender x Education |  |  |  |  |  |  |
| Men (low) | HRS | 21.83 | 8.43 | 1.01 | 9.44 | 12.39 |
|  |  | (21.43 to 22.12) | (8.17 to 8.70) | (0.92 to 1.09) | (9.15 to 9.72) | (12.01 to 12.70) |
|  | WB | 22.56 | 8.61 | 0.98 | 9.58 | 12.98 |
|  |  | (21.80 to 22.96) | (8.21 to 8.96) | (0.85 to 1.09) | (9.14 to 9.94) | (12.31 to 13.42) |
|  | EBB | 22.16 | 7.76 | 0.82 | 8.59 | 13.57 |
|  |  | (21.34 to 22.67) | (7.43 to 8.10) | (0.71 to 0.92) | (8.22 to 8.92) | (12.84 to 14.10) |
| Women (low) | HRS | 23.68 | 6.80 | 0.81 | 7.60 | 16.08 |
|  |  | (23.33 to 23.92) | (6.57 to 7.04) | (0.73 to 0.88) | (7.36 to 7.85) | (15.71 to 16.35) |
|  | WB | 24.08 | 6.65 | 0.81 | 7.45 | 16.62 |
|  |  | (23.56 to 24.42) | (6.36 to 6.93) | (0.71 to 0.89) | (7.15 to 7.74) | (16.11 to 16.99) |
|  | EBB | 24.08 | 6.54 | 0.81 | 7.35 | 16.73 |
|  |  | (23.45 to 24.55) | (6.26 to 6.84) | (0.71 to 0.91) | (7.07 to 7.63) | (16.08 to 17.21) |
| Men (high) | HRS | 23.78 | 10.49 | 1.09 | 11.58 | 12.19 |
|  |  | (23.32 to 24.13) | (10.12 to 10.80) | (0.99 to 1.21) | (11.20 to 11.89) | (11.77 to 12.56) |
|  | WB | 24.3 | 10.56 | 1.11 | 11.67 | 12.64 |
|  |  | (23.67 to 24.71) | (10.09 to 10.96) | (0.96 to 1.28) | (11.15 to 12.07) | (12.04 to 13.09) |
|  | EBB | 24.41 | 10.35 | 1.01 | 11.36 | 13.05 |
|  |  | (23.78 to 24.81) | (9.94 to 10.70) | (0.88 to 1.15) | (10.90 to 11.73) | (12.44 to 13.50) |
| Women (high) | HRS | 25.28 | 8.81 | 0.91 | 9.73 | 15.56 |
|  |  | (24.89 to 25.55) | (8.50 to 9.10) | (0.82 to 1.01) | (9.38 to 10.04) | (15.11 to 15.92) |
|  | WB | 25.71 | 8.64 | 1.00 | 9.65 | 16.06 |
|  |  | (25.18 to 26.02) | (8.30 to 8.96) | (0.88 to 1.13) | (9.26 to 9.99) | (15.51 to 16.46) |
|  | EBB | 25.73 | 9.10 | 1.04 | 10.14 | 15.59 |
|  |  | (25.14 to 26.08) | (8.77 to 9.42) | (0.92 to 1.16) | (9.78 to 10.45) | (15.02 to 15.99) |

Supplementary Table 3. Sensitivity analysis of life expectancies between ages 51 and 70 years by cohort, gender, and level of education

| Variables | Cohort | Life expectancy | In work and health | In work and unhealthy | In work | Not in work |
| --- | --- | --- | --- | --- | --- | --- |
| Total | HRS | 17.15 | 8.16 | 0.93 | 9.09 | 9.09 |
|  |  | (17.03 to 17.24) | (8.02 to 8.31) | (0.88 to 0.98) | (8.94 to 9.24) | (8.94 to 9.24) |
|  | WB | 17.43 | 8.31 | 0.94 | 9.25 | 9.25 |
|  |  | (17.28 to 17.55) | (8.11 to 8.49) | (0.89 to 1.01) | (9.05 to 9.44) | (9.05 to 9.44) |
|  | EBB | 17.39 | 8.58 | 0.96 | 9.54 | 9.54 |
|  |  | (17.25 to 17.49) | (8.42 to 8.77) | (0.90 to 1.01) | (9.35 to 9.71) | (9.35 to 9.71) |
| Gender |  |  |  |  |  |  |
| Men | HRS | 16.85 | 8.92 | 1.01 | 9.93 | 9.93 |
|  |  | (16.67 to 17.00) | (8.71 to 9.12) | (0.95 to 1.08) | (9.73 to 10.13) | (9.73 to 10.13) |
|  | WB | 17.13 | 9.34 | 0.99 | 10.33 | 10.33 |
|  |  | (16.86 to 17.32) | (9.02 to 9.62) | (0.90 to 1.09) | (10.03 to 10.61) | (10.03 to 10.61) |
|  | EBB | 17.11 | 9.20 | 0.92 | 10.12 | 10.12 |
|  |  | (16.86 to 17.28) | (8.92 to 9.45) | (0.83 to 1.01) | (9.84 to 10.37) | (9.84 to 10.37) |
| Women | HRS | 17.43 | 7.50 | 0.87 | 8.37 | 8.37 |
|  |  | (17.28 to 17.53) | (7.32 to 7.68) | (0.80 to 0.94) | (8.18 to 8.55) | (8.18 to 8.55) |
|  | WB | 17.62 | 7.71 | 0.92 | 8.63 | 8.63 |
|  |  | (17.43 to 17.73) | (7.49 to 7.91) | (0.83 to 0.99) | (8.39 to 8.83) | (8.39 to 8.83) |
|  | EBB | 17.60 | 8.11 | 0.99 | 9.10 | 9.10 |
|  |  | (17.41 to 17.72) | (7.88 to 8.32) | (0.91 to 1.08) | (8.86 to 9.30) | (8.86 to 9.30) |
| Education |  |  |  |  |  |  |
| Low | HRS | 16.92 | 7.38 | 0.91 | 8.29 | 8.29 |
|  |  | (16.76 to 17.05) | (7.20 to 7.55) | (0.85 to 0.97) | (8.10 to 8.47) | (8.10 to 8.47) |
|  | WB | 17.15 | 7.29 | 0.87 | 8.16 | 8.16 |
|  |  | (16.89 to 17.31) | (7.04 to 7.51) | (0.79 to 0.95) | (7.90 to 8.39) | (7.90 to 8.39) |
|  | EBB | 16.97 | 7.12 | 0.86 | 7.98 | 7.98 |
|  |  | (16.72 to 17.14) | (6.86 to 7.35) | (0.78 to 0.94) | (7.72 to 8.22) | (7.72 to 8.22) |
| High | HRS | 17.54 | 9.45 | 0.98 | 10.43 | 10.43 |
|  |  | (17.38 to 17.67) | (9.23 to 9.67) | (0.91 to 1.07) | (10.21 to 10.66) | (10.21 to 10.66) |
|  | WB | 17.76 | 9.45 | 1.03 | 10.48 | 10.48 |
|  |  | (17.57 to 17.90) | (9.19 to 9.72) | (0.93 to 1.13) | (10.22 to 10.76) | (10.22 to 10.76) |
|  | EBB | 17.73 | 9.75 | 1.05 | 10.80 | 10.80 |
|  |  | (17.53 to 17.85) | (9.52 to 9.98) | (0.96 to 1.14) | (10.56 to 11.05) | (10.56 to 11.05) |
| Gender x Education |  |  |  |  |  |  |
| Men (low) | HRS | 16.56 | 8.13 | 0.99 | 9.12 | 9.12 |
|  |  | (16.32 to 16.71) | (7.90 to 8.35) | (0.91 to 1.07) | (8.89 to 9.34) | (8.89 to 9.34) |
|  | WB | 16.82 | 8.34 | 0.95 | 9.28 | 9.28 |
|  |  | (16.41 to 17.02) | (7.99 to 8.65) | (0.83 to 1.04) | (8.91 to 9.59) | (8.91 to 9.59) |
|  | EBB | 16.57 | 7.7 | 0.82 | 8.52 | 8.52 |
|  |  | (16.20 to 16.77) | (7.40 to 7.98) | (0.72 to 0.92) | (8.20 to 8.81) | (8.20 to 8.81) |
| Women (low) | HRS | 17.23 | 6.81 | 0.84 | 7.65 | 7.65 |
|  |  | (17.06 to 17.34) | (6.59 to 7.01) | (0.77 to 0.91) | (7.44 to 7.87) | (7.44 to 7.87) |
|  | WB | 17.34 | 6.76 | 0.83 | 7.60 | 7.60 |
|  |  | (17.10 to 17.49) | (6.49 to 7.03) | (0.74 to 0.91) | (7.32 to 7.86) | (7.32 to 7.86) |
|  | EBB | 17.24 | 6.75 | 0.88 | 7.62 | 7.62 |
|  |  | (16.98 to 17.40) | (6.48 to 7.03) | (0.77 to 0.97) | (7.33 to 7.90) | (7.33 to 7.90) |
| Men (high) | HRS | 17.28 | 10.05 | 1.04 | 11.09 | 11.09 |
|  |  | (17.06 to 17.46) | (9.75 to 10.30) | (0.95 to 1.14) | (10.00 to 11.34) | (10.00 to 11.34) |
|  | WB | 17.47 | 10.3 | 1.04 | 11.34 | 11.34 |
|  |  | (17.15 to 17.67) | (9.92 to 10.62) | (0.92 to 1.17) | (10.95 to 11.64) | (10.95 to 11.64) |
|  | EBB | 17.5 | 10.23 | 0.99 | 11.22 | 11.22 |
|  |  | (17.23 to 17.65) | (9.89 to 10.52) | (0.89 to 1.12) | (10.87 to 11.49) | (10.87 to 11.49) |
| Women (high) | HRS | 17.79 | 8.82 | 0.92 | 9.75 | 9.75 |
|  |  | (17.61 to 17.91) | (8.57 to 9.06) | (0.84 to 1.02) | (9.46 to 10.01) | (9.46 to 10.01) |
|  | WB | 17.96 | 8.88 | 1.01 | 9.90 | 9.90 |
|  |  | (17.74 to 18.08) | (8.58 to 9.15) | (0.90 to 1.12) | (9.57 to 10.18) | (9.57 to 10.18) |
|  | EBB | 17.93 | 9.34 | 1.10 | 10.45 | 10.45 |
|  |  | (17.72 to 18.05) | (9.05 to 9.60) | (0.99 to 1.22) | (10.13 to 10.72) | (10.13 to 10.72) |

Supplementary Table 4. Sensitivity analysis of life expectancies between ages 51 and 101 years by cohort, gender, and level of education

| Variables | Cohort | Life expectancy | In work and healthy | In work and unhealthy | In work | Not in work |
| --- | --- | --- | --- | --- | --- | --- |
| Total | HRS | 29.14 | 9.39 | 1.14 | 10.53 | 18.61 |
|  |  | (28.63 to 29.61) | (9.22 to 9.58) | (1.08 to 1.20) | (10.32 to 10.72) | (18.11 to 19.12) |
|  | WB | 31.5 | 9.41 | 1.22 | 10.64 | 20.87 |
|  |  | (30.15 to 32.71) | (9.15 to 9.62) | (1.14 to 1.33) | (10.38 to 10.87) | (19.58 to 22.02) |
|  | EBB | 32.77 | 9.50 | 1.21 | 10.71 | 22.06 |
|  |  | (30.41 to 34.77) | (9.27 to 9.73) | (1.10 to 1.33) | (10.44 to 10.96) | (19.68 to 24.09) |
| Gender |  |  |  |  |  |  |
| Men | HRS | 27.14 | 10.33 | 1.26 | 11.59 | 15.56 |
|  |  | (26.51 to 27.68) | (10.05 to 10.60) | (1.17 to 1.35) | (11.31 to 11.85) | (14.98 to 16.07) |
|  | WB | 29.26 | 10.65 | 1.30 | 11.95 | 17.31 |
|  |  | (27.85 to 30.44) | (10.22 to 11.02) | (1.17 to 1.45) | (11.51 to 12.33) | (16.08 to 18.52) |
|  | EBB | 30.42 | 10.21 | 1.17 | 11.38 | 19.04 |
|  |  | (28.19 to 32.55) | (9.84 to 10.52) | (1.03 to 1.32) | (10.98 to 11.74) | (16.84 to 21.12) |
| Women | HRS | 31.18 | 8.56 | 1.05 | 9.62 | 21.56 |
|  |  | (30.37 to 31.83) | (8.31 to 8.78) | (0.97 to 1.14) | (9.35 to 9.84) | (20.78 to 22.18) |
|  | WB | 33.46 | 8.67 | 1.16 | 9.83 | 23.63 |
|  |  | (31.84 to 34.76) | (8.36 to 8.94) | (1.05 to 1.28) | (9.49 to 10.08) | (22.05 to 24.92) |
|  | EBB | 34.91 | 8.95 | 1.24 | 10.19 | 24.71 |
|  |  | (32.22 to 36.99) | (8.65 to 9.20) | (1.10 to 1.40) | (9.86 to 10.47) | (22.10 to 26.82) |
| Education |  |  |  |  |  |  |
| Low | HRS | 27.72 | 8.38 | 1.07 | 9.45 | 18.27 |
|  |  | (27.13 to 28.25) | (8.14 to 8.59) | (1.01 to 1.14) | (9.21 to 9.67) | (17.71 to 18.74) |
|  | WB | 29.64 | 8.12 | 1.07 | 9.19 | 20.45 |
|  |  | (28.24 to 30.89) | (7.81 to 8.39) | (0.97 to 1.19) | (8.86 to 9.49) | (19.12 to 21.65) |
|  | EBB | 30.46 | 7.76 | 1.02 | 8.78 | 21.68 |
|  |  | (27.96 to 32.64) | (7.45 to 8.01) | (0.91 to 1.16) | (8.46 to 9.07) | (19.28 to 23.86) |
| High | HRS | 31.60 | 11.11 | 1.26 | 12.37 | 19.23 |
|  |  | (30.77 to 32.28) | (10.80 to 11.41) | (1.16 to 1.37) | (12.03 to 12.70) | (18.44 to 19.90) |
|  | WB | 33.77 | 10.87 | 1.40 | 12.27 | 21.50 |
|  |  | (32.33 to 35.06) | (10.52 to 11.21) | (1.26 to 1.54) | (11.86 to 12.63) | (20.10 to 22.78) |
|  | EBB | 34.70 | 10.93 | 1.38 | 12.31 | 22.39 |
|  |  | (32.21 to 26.80) | (10.60 to 11.22) | (1.24 to 1.57) | (11.93 to 12.65) | (20.06 to 24.44) |
| Gender x Education |  |  |  |  |  |  |
| Men (low) | HRS | 25.36 | 9.22 | 1.18 | 10.4 | 14.96 |
|  |  | (24.63 to 25.91) | (8.91 to 9.51) | (1.09 to 1.27) | (10.07 to 10.72) | (14.34 to 15.49) |
|  | WB | 27.29 | 9.31 | 1.18 | 10.49 | 16.80 |
|  |  | (25.68 to 28.61) | (8.87 to 9.70) | (1.02 to 1.31) | (9.98 to 10.91) | (15.33 to 18.08) |
|  | EBB | 27.73 | 8.4 | 0.98 | 9.38 | 18.36 |
|  |  | (25.10 to 30.00) | (8.00 to 8.75) | (0.84 to 1.11) | (8.92 to 9.75) | (15.87 to 20.56) |
| Women (low) | HRS | 29.65 | 11.93 | 1.35 | 13.28 | 16.36 |
|  |  | (28.71 to 30.42) | (11,50 to 12.29) | (1.23 to 1.49) | (12.80 to 13.65) | (15.53 to 17.08) |
|  | WB | 31.47 | 11.94 | 1.44 | 13.38 | 18.09 |
|  |  | (29.69 to 32.82) | (11.36 to 12.39) | (1.25 to 1.65) | (12.70 to 13.85) | (16.47 to 19.38) |
|  | EBB | 32.46 | 11.50 | 1.32 | 12.81 | 19.65 |
|  |  | (29.65 to 34.74) | (11.00 to 11.87) | (1.13 to 1.54) | (12.23 to 13.65) | (16.99 to 21.90) |
| Men (high) | HRS | 29.87 | 7.69 | 1.00 | 8.69 | 21.18 |
|  |  | (29.05 to 30.54) | (7.43 to 7.96) | (0.91 to 1.08) | (8.41 to 8.95) | (20.36 to 21.84) |
|  | WB | 31.54 | 7.49 | 1.01 | 8.50 | 23.04 |
|  |  | (29.81 to 32.93) | (7.17 to 7.81) | (0.88 to 1.12) | (8.16 to 8.81) | (21.35 to 24.36) |
|  | EBB | 32.72 | 7.36 | 1.04 | 8.40 | 24.33 |
|  |  | (29.84 to 34.96) | (7.04 to 7.67) | (0.91 to 1.18) | (8.06 to 8.71) | (21.43 to 26.48) |
| Women (high) | HRS | 33.88 | 10.28 | 1.17 | 11.45 | 22.44 |
|  |  | (32.80 to 34.75) | (9.90 to 10.61) | (1.05 to 1.29) | (11.02 to 11.84) | (21.38 to 23.25) |
|  | WB | 35.97 | 10.13 | 1.35 | 11.48 | 24.49 |
|  |  | (33.99 to 37.47) | (9.71 to 10.49) | (1.18 to 1.53) | (10.99 to 11.88) | (22.59 to 25.97) |
|  | EBB | 36.94 | 10.43 | 1.45 | 11.87 | 25.07 |
|  |  | (34.03 to 39.08) | (10.04 to 10.78) | (1.25 to 1.65) | (11.40 to 12.26) | (22.30 to 27.13) |

Supplementary Table 5. Sensitivity analysis of life expectancies between ages 51 and 80 years for Caucasians/Whites by cohort, gender, and level of education

| Variables | Cohort | Life expectancy | In work and healthy | In work and unhealthy | In work | Not in work |
| --- | --- | --- | --- | --- | --- | --- |
| Total | HRS | 23.73 | 9.17 | 1.20 | 10.38 | 13.35 |
|  |  | (23.48 to 23.93) | (8.99 to 9.39) | (1.13 to 1.26) | (10.14 to 10.59) | (13.09 to 13.60) |
|  | WB | 24.51 | 9.22 | 1.22 | 10.44 | 14.07 |
|  |  | (24.16 to 24.78) | (8.96 to 9.45) | (1.14 to 1.30) | (10.17 to 10.68) | (13.72 to 14.34) |
|  | EBB | 24.43 | 9.42 | 1.27 | 10.69 | 13.74 |
|  |  | (23.94 to 24.87) | (9.12 to 9.72) | (1.15 to 1.39) | (10.36 to 11.00) | (13.19 to 14.25) |
| Gender |  |  |  |  |  |  |
| Men | HRS | 23.02 | 10.17 | 1.31 | 11.48 | 11.53 |
|  |  | (22.63 to 23.34) | (9.86 to 10.47) | (1.22 to 1.42) | (11.18 to 11.78) | (11.17 to 11.86) |
|  | WB | 23.60 | 10.55 | 1.21 | 11.77 | 11.84 |
|  |  | (23.05 to 24.08) | (10.10 to 10.96) | (1.08 to 1.36) | (11.31 to 12.19) | (11.29 to 12.27) |
|  | EBB | 23.48 | 10.23 | 1.28 | 11.50 | 11.97 |
|  |  | (22.73 to 24.02) | (9.76 to 10.60) | (1.11 to 1.43) | (11.03 to 11.93) | (11.30 to 12.53) |
| Women | HRS | 24.37 | 8.28 | 1.10 | 9.39 | 14.99 |
|  |  | (24.01 to 24.64) | (8.01 to 8.53) | (1.01 to 1.20) | (9.09 to 9.65) | (14.61 to 15.31) |
|  | WB | 25.11 | 8.45 | 1.22 | 9.67 | 15.45 |
|  |  | (24.66 to 25.41) | (8.13 to 8.73) | (1.10 to 1.33) | (9.34 to 9.94) | (14.98 to 15.82) |
|  | EBB | 25.32 | 8.82 | 1.26 | 10.08 | 15.24 |
|  |  | (24.60 to 25.77) | (8.45 to 9.14) | (1.10 to 1.41) | (9.68 to 10.41) | (14.56 to 15.76) |
| Education |  |  |  |  |  |  |
| Low | HRS | 23.06 | 8.13 | 1.16 | 9.29 | 13.77 |
|  |  | (22.69 to 23.37) | (7.86 to 8.37) | (1.08 to 1.25) | (9.01 to 9.56) | (13.40 to 14.10) |
|  | WB | 23.59 | 7.86 | 1.14 | 9.00 | 14.60 |
|  |  | (23.05 to 23.99) | (7.53 to 8.16) | (1.03 to 1.26) | (8.64 to 9.32) | (14.05 to 15.04) |
|  | EBB | 23.03 | 7.39 | 1.16 | 8.55 | 14.48 |
|  |  | (21.75 to 23.67) | (7.00 to 7.74) | (0.98 to 1.33) | (8.08 to 8.93) | (13.43 to 15.13) |
| High | HRS | 24.66 | 10.62 | 1.26 | 11.87 | 12.79 |
|  |  | (24.27 to 24.98) | (10.30 to 10.92) | (1.14 to 1.37) | (11.53 to 12.21) | (12.38 to 13.14) |
|  | WB | 25.39 | 10.51 | 1.29 | 11.80 | 13.59 |
|  |  | (24.95 to 25.73) | (10.14 to 10.86) | (1.16 to 1.43) | (11.39 to 12.16) | (13.08 to 13.96) |
|  | EBB | 25.29 | 10.66 | 1.33 | 11.99 | 13.30 |
|  |  | (24.72 to 25.68) | (10.27 to 10.99) | (1.19 to 1.48) | (11.58 to 12.35) | (12.73 to 13.78) |
| Gender x Education |  |  |  |  |  |  |
| Men (low) | HRS | 22.08 | 9.03 | 1.29 | 10.33 | 11.76 |
|  |  | (21.59 to 22.42) | (8.69 to 9.37) | (1.17 to 1.41) | (9.95 to 10.68) | (11.31 to 12.12) |
|  | WB | 22.50 | 9.13 | 1.16 | 10.29 | 12.20 |
|  |  | (21.59 to 23.01) | (8.66 to 9.58) | (0.99 to 1.31) | (9.75 to 10.77) | (11.40 to 12.76) |
|  | EBB | 21.55 | 8.07 | 1.18 | 9.25 | 12.30 |
|  |  | (20.33 to 22.28) | (7.58 to 8.54) | (0.99 to 1.36) | (8.72 to 9.74) | (11.17 to 13.06) |
| Women (low) | HRS | 24.07 | 11.44 | 1.34 | 12.78 | 11.29 |
|  |  | (23.56 to 24.46) | (11.00 to 11.82) | (1.21 to 1.48) | (12.31 to 13.16) | (10.82 to 11.68) |
|  | WB | 24.54 | 11.65 | 1.27 | 12.91 | 11.62 |
|  |  | (23.83 to 24.99) | (11.08 to 12.13) | (1.10 to 1.46) | (12.28 to 13.41) | (10.97 to 12.12) |
|  | EBB | 24.45 | 11.26 | 1.33 | 12.59 | 11.86 |
|  |  | (23.65 to 25.01) | (10.70 to 11.71) | (1.15 to 1.53) | (11.96 to 13.12) | (11.07 to 12.43) |
| Men (high) | HRS | 23.79 | 7.41 | 1.08 | 8.49 | 15.30 |
|  |  | (23.35 to 24.07) | (7.11 to 7.72) | (0.97 to 1.18) | (8.17 to 8.81) | (14.84 to 15.64) |
|  | WB | 24.24 | 7.25 | 1.12 | 8.37 | 15.87 |
|  |  | (23.63 to 24.63) | (6.91 to 7.59) | (0.98 to 1.25) | (8.02 to 8.71) | (15.28 to 16.31) |
|  | EBB | 24.13 | 7.02 | 1.14 | 8.16 | 15.98 |
|  |  | (23.17 to 24.78) | (6.65 to 7.44) | (0.95 to 1.32) | (7.74 to 8.57) | (14.99 to 16.63) |
| Women (high) | HRS | 25.40 | 9.74 | 1.17 | 10.90 | 14.50 |
|  |  | (24.95 to 25.71) | (9.35 to 10.09) | (1.03 to 1.30) | (10.57 to 11.43) | (13.97 to 14.94) |
|  | WB | 26.02 | 9.73 | 1.31 | 11.04 | 14.98 |
|  |  | (25.46 to 26.34) | (9.31 to 10.14) | (1.14 to 1.48) | (10.57 to 11.43) | (14.36 to 15.44) |
|  | EBB | 26.14 | 10.13 | 1.34 | 11.47 | 14.67 |
|  |  | (25.39 to 26.58) | (9.66 to 10.57) | (1.15 to 1.52) | (10.92 to 11.93) | (13.92 to 15.20) |

Supplementary Table 6. Sensitivity analysis of life expectancies between ages 51 and 80 years by cohort, gender, and level of education using MiddleRiemann method for numerical approximation

| Variables | Cohort | Life expectancy | In work and healthy | In work and unhealthy | In work | Not in work |
| --- | --- | --- | --- | --- | --- | --- |
| Total | HRS | 23.46 | 8.92 | 1.08 | 10.00 | 13.45 |
|  |  | (23.26 to 23.64) | (8.77 to 9.11) | (1.02 to 1.13) | (9.81 to 10.18) | (13.23 to 13.66) |
|  | WB | 24.22 | 8.97 | 1.13 | 10.10 | 14.12 |
|  |  | (23.91 to 24.48) | (8.74 to 9.17) | (1.06 to 1.21) | (9.86 to 10.31) | (13.81 to 14.39) |
|  | EBB | 24.24 | 9.05 | 1.12 | 10.17 | 14.07 |
|  |  | (23.86 to 24.56) | (8.83 to 9.27) | (1.04 to 1.20) | (9.93 to 10.39) | (13.66 to 14.46) |
| Gender |  |  |  |  |  |  |
| Men | HRS | 22.61 | 9.82 | 1.18 | 11.01 | 11.60 |
|  |  | (22.27 to 22.90) | (9.57 to 10.08) | (1.10 to 1.27) | (10.76 to 11.26) | (11.30 to 11.88) |
|  | WB | 23.39 | 10.18 | 1.20 | 11.39 | 12.00 |
|  |  | (22.90 to 23.79) | (9.80 to 10.54) | (1.09 to 1.33) | (10.99 to 11.75) | (11.53 to 12.39) |
|  | EBB | 23.44 | 9.76 | 1.09 | 10.85 | 12.59 |
|  |  | (22.88 to 23.86) | (9.41 to 10.06) | (0.97 to 1.21) | (10.49 to 11.17) | (12.06 to 13.03) |
| Women | HRS | 24.21 | 8.14 | 1.00 | 9.14 | 15.07 |
|  |  | (23.92 to 24.44) | (7.91 to 8.35) | (0.92 to 1.08) | (8.90 to 9.36) | (14.76 to 15.34) |
|  | WB | 24.78 | 8.24 | 1.08 | 9.32 | 15.46 |
|  |  | (24.37 to 25.05) | (7.96 to 8.50) | (0.98 to 1.17) | (9.03 to 9.57) | (15.06 to 15.79) |
|  | EBB | 24.90 | 8.51 | 1.15 | 9.66 | 15.23 |
|  |  | (24.38 to 25.26) | (8.24 to 8.76) | (1.03 to 1.26) | (9.36 to 9.90) | (14.76 to 15.62) |
| Education |  |  |  |  |  |  |
| Low | HRS | 22.83 | 7.95 | 1.03 | 8.98 | 13.85 |
|  |  | (22.55 to 23.09) | (7.73 to 8.15) | (0.96 to 1.09) | (8.76 to 9.19) | (13.57 to 14.13) |
|  | WB | 23.46 | 7.73 | 1.01 | 8.74 | 14.73 |
|  |  | (22.96 to 23.80) | (7.44 to 7.99) | (0.91 to 1.10) | (8.43 to 9.02) | (14.25 to 15.09) |
|  | EBB | 23.23 | 7.36 | 0.96 | 8.32 | 14.92 |
|  |  | (22.66 to 23.67) | (7.08 to 7.61) | (0.86 to 1.07) | (8.03 to 8.60) | (14.38 to 15.35) |
| High | HRS | 24.52 | 10.55 | 1.18 | 11.73 | 12.79 |
|  |  | (24.17 to 24.80) | (10.26 to 10.83) | (1.08 to 1.28) | (11.42 to 12.03) | (12.43 to 13.09) |
|  | WB | 25.10 | 10.36 | 1.27 | 11.63 | 13.47 |
|  |  | (24.68 to 25.41) | (10.03 to 10.69) | (1.14 to 1.39) | (11.27 to 11.97) | (12.99 to 13.80) |
|  | EBB | 25.08 | 10.43 | 1.27 | 11.70 | 13.38 |
|  |  | (24.62 to 25.40) | (10.13 to 10.71) | (1.15 to 1.38) | (11.37 to 11.98) | (12.91 to 13.76) |
| Gender x Education |  |  |  |  |  |  |
| Men (low) | HRS | 21.82 | 8.80 | 1.14 | 9.94 | 11.88 |
|  |  | (21.41 to 22.11) | (8.53 to 9.08) | (1.05 to 1.23) | (9.63 to 10.23) | (11.51 to 12.19) |
|  | WB | 22.52 | 8.91 | 1.11 | 10.01 | 12.5 |
|  |  | (21.75 to 22.92) | (8.49 to 9.28) | (0.97 to 1.22) | (9.54 to 10.42) | (11.84 to 12.97) |
|  | EBB | 22.14 | 7.98 | 0.92 | 8.90 | 13.24 |
|  |  | (21.33 to 22.67) | (7.63 to 8.32) | (0.81 to 1.04) | (8.52 to 9.27) | (12.51 to 13.79) |
| Women (low) | HRS | 23.68 | 7.30 | 0.95 | 8.25 | 15.44 |
|  |  | (23.33 to 23.93) | (7.05 to 7.55) | (0.86 to 1.03) | (7.99 to 8.50) | (15.08 to 15.72) |
|  | WB | 24.06 | 7.12 | 0.95 | 8.07 | 15.98 |
|  |  | (23.53 to 24.39) | (6.83 to 7.43) | (0.83 to 1.05) | (7.75 to 8.37) | (15.48 to 16.36) |
|  | EBB | 24.02 | 6.95 | 0.98 | 7.93 | 16.09 |
|  |  | (23.38 to 24.49) | (6.66 to 7.26) | (0.86 to 1.10) | (7.63 to 8.22) | (15.44 to 16.58) |
| Men (high) | HRS | 23.79 | 11.30 | 1.26 | 12.57 | 11.22 |
|  |  | (23.34 to 24.14) | (10.91 to 11.63) | (1.15 to 1.39) | (12.15 to 12.90) | (10.81 to 11.58) |
|  | WB | 24.29 | 11.40 | 1.31 | 12.71 | 11.58 |
|  |  | (23.67 to 24.69) | (10.89 to 11.83) | (1.15 to 1.48) | (12.14 to 13.13) | (11.01 to 12.02) |
|  | EBB | 24.37 | 11.01 | 1.21 | 12.22 | 12.15 |
|  |  | (23.76 to 24.80) | (10.56 to 11.38) | (1.07 to 1.37) | (11.72 to 12.62) | (11.54 to 12.61) |
| Women (high) | HRS | 25.24 | 9.76 | 1.10 | 10.86 | 14.38 |
|  |  | (24.85 to 25.51) | (9.42 to 10.07) | (0.99 to 1.20) | (10.48 to 11.21) | (13.93 to 14.76) |
|  | WB | 25.70 | 9.64 | 1.23 | 10.87 | 14.82 |
|  |  | (25.17 to 26.00) | (9.25 to 9.99) | (1.09 to 1.38) | (10.44 to 11.25) | (14.27 to 15.25) |
|  | EBB | 25.70 | 9.94 | 1.33 | 11.27 | 14.43 |
|  |  | (25.11 to 26.07) | (9.58 to 10.29) | (1.17 to 1.48) | (10.87 to 11.64) | (13.84 to 14.85) |

Supplementary Table 7. Sensitivity analysis of life expectancies between ages 51 and 80 years by cohort, gender, and level of education using Simpson method for numerical approximation

| Variables | Cohort | Life expectancy | In work and healthy | In work and unhealthy | In work | Not in work |
| --- | --- | --- | --- | --- | --- | --- |
| Total | HRS | 23.33 | 8.90 | 1.08 | 10.03 | 13.35 |
|  |  | (23.13 to 23.51) | (8.75 to 9.09) | (1.02 to 1.13) | (9.84 to 10.21) | (13.13 to 13.55) |
|  | WB | 24.22 | 8.97 | 1.13 | 10.13 | 14.12 |
|  |  | (23.91 to 24.48) | (8.74 to 9.17) | (1.06 to 1.21) | (9.90 to 10.35) | (13.81 to 14.39) |
|  | EBB | 24.10 | 9.04 | 1.12 | 10.21 | 13.94 |
|  |  | (23.72 to 24.41) | (8.82 to 9.26) | (1.03 to 1.20) | (9.96 to 10.42) | (13.54 to 14.32) |
| Gender |  |  |  |  |  |  |
| Men | HRS | 22.50 | 9.80 | 1.18 | 11.03 | 11.51 |
|  |  | (22.16 to 22.78) | (9.55 to 10.06) | (1.10 to 1.26) | (10.78 to 11.29) | (11.21 to 11.79) |
|  | WB | 23.26 | 10.16 | 1.20 | 11.42 | 11.90 |
|  |  | (22.78 to 23.66) | (9.78 to 10.52) | (1.08 to 1.32) | (11.02 to 11.78) | (11.43 to 12.28) |
|  | EBB | 23.31 | 9.75 | 1.09 | 10.88 | 12.47 |
|  |  | (22.76 to 23.72) | (9.40 to 10.05) | (0.97 to 1.20) | (10.52 to 11.20) | (11.96 to 12.90) |
| Women | HRS | 24.07 | 8.13 | 1 | 9.17 | 14.95 |
|  |  | (23.78 to 24.30) | (7.90 to 8.33) | (0.92 to 1.07) | (8.92 to 9.38) | (14.64 to 15.21) |
|  | WB | 24.63 | 8.23 | 1.08 | 9.35 | 15.32 |
|  |  | (24.23 to 24.89) | (7.95 to 8.49) | (0.98 to 1.17) | (9.05 to 9.60) | (14.93 to 15.65) |
|  | EBB | 24.74 | 8.51 | 1.15 | 9.69 | 15.09 |
|  |  | (24.23 to 25.10) | (8.23 to 8.75) | (1.03 to 1.26) | (9.39 to 9.93) | (14.62 to 15.46) |
| Education |  |  |  |  |  |  |
| Low | HRS | 22.72 | 7.93 | 1.03 | 9.00 | 13.76 |
|  |  | (22.43 to 22.97) | (7.72 to 8.13) | (0.96 to 1.09) | (8.78 to 9.21) | (13.47 to 14.03) |
|  | WB | 23.34 | 7.72 | 1.00 | 8.77 | 14.61 |
|  |  | (22.84 to 23.67) | (7.43 to 7.98) | (0.91 to 1.10) | (8.45 to 9.05) | (14.15 to 14.98) |
|  | EBB | 23.10 | 7.35 | 0.96 | 8.34 | 14.80 |
|  |  | (22.54 to 23.53) | (7.07 to 7.60) | (0.86 to 1.07) | (8.05 to 8.63) | (14.27 to 15.22) |
| High | HRS | 24.38 | 10.53 | 1.18 | 11.76 | 12.67 |
|  |  | (24.03 to 24.65) | (10.24 to 10.81) | (1.08 to 1.28) | (11.45 to 12.06) | (12.31 to 12.97) |
|  | WB | 24.94 | 10.34 | 1.26 | 11.66 | 13.33 |
|  |  | (24.53 to 25.25) | (10.01 to 10.67) | (1.14 to 1.39) | (11.30 to 12.00) | (12.87 to 13.66) |
|  | EBB | 24.92 | 10.42 | 1.27 | 11.74 | 13.24 |
|  |  | (24.47 to 25.24) | (10.12 to 10.70) | (1.15 to 1.38) | (11.40 to 12.02) | (12.77 to 13.61) |
| Gender x Education |  |  |  |  |  |  |
| Men (low) | HRS | 21.72 | 8.79 | 1.13 | 9.97 | 11.80 |
|  |  | (21.31 to 22.01) | (8.51 to 9.06) | (1.04 to 1.22) | (9.66 to 10.26) | (11.44 to 12.11) |
|  | WB | 22.41 | 8.89 | 1.11 | 10.04 | 12.41 |
|  |  | (21.65 to 22.80) | (8.48 to 9.27) | (0.96 to 1.22) | (9.57 to 10.45) | (11.75 to 12.87) |
|  | EBB | 22.03 | 7.97 | 0.92 | 8.93 | 13.14 |
|  |  | (21.23 to 22.55) | (7.62 to 8.31) | (0.81 to 1.03) | (8.55 to 9.30) | (12.42 to 13.68) |
| Women (low) | HRS | 23.55 | 7.28 | 0.95 | 8.27 | 15.32 |
|  |  | (23.21 to 23.80) | (7.04 to 7.54) | (0.86 to 1.02) | (8.01 to 8.53) | (14.96 to 15.60) |
|  | WB | 23.92 | 7.11 | 0.95 | 8.10 | 15.86 |
|  |  | (23.40 to 24.25) | (6.81 to 7.42) | (0.83 to 1.05) | (7.77 to 8.40) | (15.36 to 16.23) |
|  | EBB | 23.87 | 6.94 | 0.97 | 7.96 | 15.96 |
|  |  | (23.25 to 24.33) | (6.65 to 7.25) | (0.85 to 1.09) | (7.66 to 8.25) | (15.31 to 16.43) |
| Men (high) | HRS | 23.66 | 11.28 | 1.26 | 12.60 | 11.12 |
|  |  | (23.21 to 24.01) | (10.89 to 11.60) | (1.15 to 1.38) | (12.18 to 12.93) | (10.71 to 11.47) |
|  | WB | 24.15 | 11.38 | 1.31 | 12.74 | 11.46 |
|  |  | (23.53 to 24.55) | (10.87 to 11.81) | (1.14 to 1.48) | (12.17 to 13.16) | (10.90 to 11.91) |
|  | EBB | 24.23 | 11.00 | 1.21 | 12.26 | 12.02 |
|  |  | (23.63 to 24.65) | (10.55 to 11.37) | (1.07 to 1.37) | (11.75 to 12.66) | (11.42 to 12.48) |
| Women (high) | HRS | 25.08 | 9.74 | 1.09 | 10.89 | 14.25 |
|  |  | (24.70 to 25.35) | (9.40 to 10.05) | (0.99 to 1.20) | (10.51 to 11.24) | (13.79 to 14.63) |
|  | WB | 25.53 | 9.62 | 1.23 | 10.90 | 14.68 |
|  |  | (25.02 to 25.83) | (9.24 to 9.98) | (1.09 to 1.37) | (10.47 to 11.29) | (14.13 to 15.10) |
|  | EBB | 25.54 | 9.93 | 1.33 | 11.31 | 14.28 |
|  |  | (24.96 to 25.89) | (9.57 to 10.28) | (1.17 to 1.48) | (10.91 to 11.67) | (13.69 to 14.69) |

Supplementary Table 8. Life expectancies and working life expectancies for individuals working in good health between ages 51 and 80 by cohort, gender, and level of education

| Variables | Cohort | Life expectancy | In work and healthy | In work and unhealthy | In work | Not in work |
| --- | --- | --- | --- | --- | --- | --- |
| Total | HRS | **23.82** | **10.27** | **1.05** | **11.32** | **12.50** |
|  |  |  | (10.08 to 10.45) | (0.99 to 1.10) | (11.11 to 11.51) | (12.29 to 12.70) |
|  | WB | **24.59** | **10.29** | **1.12** | **11.42** | **13.18** |
|  |  |  | (10.06 to 10.52) | (1.04 to 1.20) | (11.16 to 11.66) | (12.87 to 13.44) |
|  | EBB | **24.69** | **10.33** | **1.07** | **11.40** | **13.29** |
|  |  |  | (10.09 to 10.54) | (0.98 to 1.15) | (11.15 to 11.64) | (12.87 to 13.68) |
| Gender |  |  |  |  |  |  |
| Men | HRS | 22.91 | 10.80 | 1.12 | 11.93 | 10.99 |
|  |  |  | (10.55 to 11.06) | (1.04 to 1.21) | (11.67 to 12.19) | (10.71 to 11.27) |
|  | WB | 23.73 | 11.11 | 1.18 | 12.29 | 11.44 |
|  |  |  | (10.73 to 11.46) | (1.06 to 1.30) | (11.91 to 12.65) | (10.99 to 11.83) |
|  | EBB | 23.93 | 10.88 | 1.04 | 11.92 | 12.01 |
|  |  |  | (10.54 to 11.19) | (0.92 to 1.15) | (11.56 to 12.24) | (11.51 to 12.44) |
| Women | HRS | 24.63 | 9.78 | 0.98 | 10.77 | 13.87 |
|  |  |  | (9.53 to 10.01) | (0.91 to 1.06) | (10.50 to 11.01) | (13.55 to 14.13) |
|  | WB | 25.17 | 9.74 | 1.08 | 10.83 | 14.35 |
|  |  |  | (9.44 to 10.00) | (0.98 to 1.18) | (10.51 to 11.09) | (13.95 to 14.68) |
|  | EBB | 25.32 | 9.89 | 1.11 | 11.00 | 14.32 |
|  |  |  | (9.60 to 10.15) | (0.99 to 1.22) | (10.68 to 11.26) | (13.84 to 14.72) |
| Education |  |  |  |  |  |  |
| Low | HRS | 23.28 | 9.49 | 1.01 | 10.50 | 12.78 |
|  |  |  | (9.24 to 9.70) | (0.94 to 1.08) | (10.24 to 10.72) | (12.47 to 13.05) |
|  | WB | 23.95 | 9.29 | 1.03 | 10.33 | 13.63 |
|  |  |  | (8.97 to 9.56) | (0.94 to 1.13) | (9.97 to 10.63) | (13.12 to 14.00) |
|  | EBB | 23.84 | 8.93 | 0.94 | 9.87 | 13.97 |
|  |  |  | (8.62 to 9.20) | (0.84 to 1.05) | (9.54 to 10.18) | (13.38 to 14.42 |
| High | HRS | 24.74 | 11.50 | 1.12 | 12.62 | 12.12 |
|  |  |  | (11.23 to 11.78) | (1.03 to 1.21) | (12.33 to 12.92) | (11.75 to 12.42) |
|  | WB | 25.33 | 11.34 | 1.22 | 12.56 | 12.77 |
|  |  |  | (11.01 to 11.66) | (1.10 to 1.35) | (12.19 to 12.90) | (12.31 to 13.10) |
|  | EBB | 25.38 | 11.38 | 1.19 | 12.57 | 12.81 |
|  |  |  | (11.08 to 11.65) | (1.07 to 1.31) | (12.23 to 12.84) | (12.37 to 13.19) |
| Gender x Education |  |  |  |  |  |  |
| Men (low) | HRS | 22.21 | 9.95 | 1.09 | 11.03 | 11.17 |
|  |  |  | (9.66 to 10.23) | (0.99 to 1.17) | (10.72 to 11.33) | (10.82 to 11.47) |
|  | WB | 22.97 | 10.04 | 1.11 | 11.14 | 11.82 |
|  |  |  | (9.60 to 10.42) | (0.96 to 1.23) | (10.67 to 11.56) | (11.14 to 12.26) |
|  | EBB | 22.81 | 9.39 | 0.90 | 10.29 | 12.52 |
|  |  |  | (9.01 to 9.76) | (0.78 to 1.01) | (9.90 to 10.65) | (11.78 to 13.06) |
| Women (low) | HRS | 24.18 | 9.11 | 0.96 | 10.07 | 14.11 |
|  |  |  | (8.85 to 9.39) | (0.87 to 1.04) | (9.80 to 10.34) | (13.74 to 14.39) |
|  | WB | 24.58 | 8.86 | 0.99 | 9.84 | 14.73 |
|  |  |  | (8.52 to 9.19) | (0.87 to 1.09) | (9.50 to 10.18) | (14.22 to 15.10) |
|  | EBB | 24.58 | 8.61 | 0.97 | 9.58 | 15.00 |
|  |  |  | (8.29 to 8.95) | (0.85 to 1.10) | (9.26 to 9.90) | (14.36 to 15.44) |
| Men (high) | HRS | 24.00 | 12.02 | 1.19 | 13.20 | 10.79 |
|  |  |  | (11.61 to 12.35) | (1.07 to 1.31) | (12.78 to 13.53) | (10.38 to 11.13) |
|  | WB | 24.50 | 12.08 | 1.26 | 13.34 | 11.16 |
|  |  |  | (11.57 to 12.53) | (1.10 to 1.42) | (12.77 to 13.76) | (10.60 to 11.62) |
|  | EBB | 24.70 | 11.85 | 1.14 | 12.99 | 11.71 |
|  |  |  | (11.39 to 12.21) | (1.00 to 1.30) | (12.47 to 13.36) | (11.10 to 12.16) |
| Women (high) | HRS | 25.50 | 10.96 | 1.05 | 12.01 | 13.49 |
|  |  |  | (10.63 to 11.30) | (0.94 to 1.16) | (11.64 to 12.35) | (13.06 to 13.85) |
|  | WB | 25.95 | 10.77 | 1.20 | 11.96 | 13.98 |
|  |  |  | (10.37 to 11.15) | (1.05 to 1.34) | (11.53 to 12.33 | (13.44 to 14.38) |
|  | EBB | 25.97 | 10.97 | 1.25 | 12.22 | 13.75 |
|  |  |  | (10.61 to 11.33) | (1.09 to 1.42) | (11.83 to 12.57) | (13.16 to 14.17) |

Supplementary Table 9. Life expectancies and working life expectancies for individuals working in poor health between ages 51 and 80 years by cohort, gender, and level of education

| Variables | Cohort | Life expectancy | In work and healthy | In work and unhealthy | In work | Not in work |
| --- | --- | --- | --- | --- | --- | --- |
| Total | HRS | **23.24** | **7.36** | **2.56** | **9.92** | **13.32** |
|  |  |  | (7.03 to 7.65) | (2.41 to 2.7) | (9.6 to 10.19) | (12.99 to 13.63) |
|  | WB | **23.86** | **7.32** | **2.51** | **9.83** | **14.03** |
|  |  |  | (6.85 to 7.72) | (2.33 to 2.72) | (9.34 to 10.22) | (13.49 to 14.44) |
|  | EBB | **23.80** | **7.23** | **2.69** | **9.93** | **13.88** |
|  |  |  | (6.75 to 7.62) | (2.48 to 2.9) | (9.46 to 10.27) | (13.25 to 14.39) |
| Gender |  |  |  |  |  |  |
| Men | HRS | 22.31 | 8.08 | 2.58 | 10.66 | 11.65 |
|  |  |  | (7.64 to 8.44) | (2.4 to 2.77) | (10.21 to 11) | (11.2 to 12.0) |
|  | WB | 22.52 | 8.28 | 2.46 | 10.74 | 11.78 |
|  |  |  | (7.54 to 8.87) | (2.18 to 2.75) | (9.91 to 11.28) | (10.96 to 12.33) |
|  | EBB | 22.75 | 7.77 | 2.58 | 10.36 | 12.40 |
|  |  |  | (7.06 to 8.34) | (2.29 to 2.86) | (9.56 to 10.84) | (11.46 to 12.96) |
| Women | HRS | 23.95 | 6.64 | 2.57 | 9.22 | 14.74 |
|  |  |  | (6.23 to 7.07) | (2.37 to 2.78) | (8.8 to 9.62) | (14.22 to 15.19) |
|  | WB | 24.54 | 6.66 | 2.52 | 9.18 | 15.36 |
|  |  |  | (6.21 to 7.14) | (2.28 to 2.75) | (8.71 to 9.62) | (14.76 to 15.86) |
|  | EBB | 24.63 | 6.81 | 2.78 | 9.59 | 15.04 |
|  |  |  | (6.32 to 7.31) | (2.49 to 3.05) | (9.08 to 10.03) | (14.28 to 15.59) |
| Education |  |  |  |  |  |  |
| Low | HRS | 22.65 | 6.55 | 2.49 | 9.04 | 13.61 |
|  |  |  | (6.16 to 6.91) | (2.31 to 2.67) | (8.66 to 9.4) | (13.17 to 13.95) |
|  | WB | 23.40 | 6.42 | 2.28 | 8.71 | 14.70 |
|  |  |  | (5.89 to 6.91) | (2.06 to 2.51) | (8.15 to 9.17) | (13.96 to 15.17) |
|  | EBB | 23.00 | 5.95 | 2.40 | 8.35 | 14.65 |
|  |  |  | (5.43 to 6.47) | (2.14 to 2.67) | (7.79 to 8.83) | (13.86 to 15.25) |
| High | HRS | 23.98 | 8.62 | 2.66 | 11.28 | 12.70 |
|  |  |  | (8.12 to 9.07) | (2.44 to 2.9) | (10.75 to 11.7) | (12.09 to 13.11) |
|  | WB | 24.63 | 8.36 | 2.78 | 11.14 | 13.49 |
|  |  |  | (7.76 to 8.88) | (2.49 to 3.08) | (10.47 to 11.6) | (12.76 to 13.98) |
|  | EBB | 24.45 | 8.30 | 2.91 | 11.21 | 13.24 |
|  |  |  | (7.68 to 8.82) | (2.61 to 3.21) | (10.53 to 11.64) | (12.45 to 13.74) |
| Gender x Education |  |  |  |  |  |  |
| Men (low) | HRS | 21.65 | 7.25 | 2.52 | 9.76 | 11.88 |
|  |  |  | (6.80 to 7.65) | (2.32 to 2.7) | (9.35 to 10.14) | (11.39 to 12.23) |
|  | WB | 22.29 | 7.45 | 2.31 | 9.76 | 12.53 |
|  |  |  | (6.72 to 8.03) | (2.01 to 2.57) | (8.99 to 10.29) | (11.58 to 13.1) |
|  | EBB | 21.69 | 6.35 | 2.31 | 8.54 | 13.03 |
|  |  |  | (5.63 to 6.97) | (2.00 to 2.57) | (8.13 to 8.94) | (11.77 to 13.67) |
| Women (low) | HRS | 23.53 | 6.03 | 2.51 | 8.54 | 14.99 |
|  |  |  | (5.63 to 6.43) | (2.31 to 2.73) | (8.13 to 8.94) | (14.46 to 15.41) |
|  | WB | 24.03 | 5.86 | 2.26 | 8.12 | 15.91 |
|  |  |  | (5.35 to 6.38) | (2.01 to 2.49) | (7.59 to 8.61) | (15.22 to 16.44) |
|  | EBB | 23.90 | 5.61 | 2.49 | 8.10 | 15.80 |
|  |  |  | (5.09 to 6.10) | (2.22 to 2.76) | (7.58 to 8.59) | (14.86 to 16.41) |
| Men (high) | HRS | 23.19 | 9.28 | 2.67 | 11.95 | 11.24 |
|  |  |  | (8.67 to 9.78) | (2.43 to 2.91) | (11.33 to 12.41) | (10.68 to 11.69) |
|  | WB | 23.47 | 9.34 | 2.69 | 12.03 | 11.44 |
|  |  |  | (8.41 to 10.06) | (2.35 to 3.03) | (11.07 to 12.66) | (10.5 to 12.1) |
|  | EBB | 23.44 | 8.72 | 2.77 | 11.49 | 11.95 |
|  |  |  | (7.88 to 9.38) | (2.46 to 3.09) | (10.56 to 12.08) | (11.02 to 12.56) |
| Women (high) | HRS | 22.28 | 7.79 | 2.68 | 10.47 | 14.11 |
|  |  |  | (7.20 to 8.31) | (2.42 to 2.92) | (9.86 to 10.97) | (13.3 to 14.65) |
|  | WB | 25.39 | 7.72 | 2.81 | 10.53 | 14.86 |
|  |  |  | (7.10 to 8.26) | (2.51 to 3.11) | (9.95 to 11.06) | (14.16 to 15.35) |
|  | EBB | 25.27 | 7.89 | 3.06 | 10.96 | 14.32 |
|  |  |  | (7.28 to 8.43) | (2.74 to 3.35) | (10.32 to 11.46) | (13.49 to 14.83) |

Supplementary Table 10. Sample characteristics by cohort

|  | HRS |  | WB |  | EBB |  |
| --- | --- | --- | --- | --- | --- | --- |
|  | N | % | N | % | N | % |
| Gender |  |  |  |  |  |  |
| Men | 2769 | 47.21% | 1434 | 40.99% | 2060 | 44.72% |
| Women | 3096 | 52.79% | 2064 | 59.01% | 2546 | 55.28% |
| Education |  |  |  |  |  |  |
| Low | 3668 | 62.54% | 1844 | 52.72% | 2104 | 45.68% |
| Men | 1623 | 27.67% | 703 | 20.10% | 875 | 19.00% |
| Women | 2045 | 34.87% | 1141 | 32.62% | 1229 | 26.68% |
| High | 2197 | 37.46% | 1654 | 47.28% | 2502 | 54.32% |
| Men | 1146 | 19.54% | 731 | 20.90% | 1185 | 25.73% |
| Women | 1051 | 17.92% | 923 | 26.39% | 1317 | 28.59% |
| Race |  |  |  |  |  |  |
| White/Caucasian | 4131 | 70.43% | 2540 | 72.61% | 2530 | 54.93% |
| Black/African American | 993 | 16.93% | 546 | 15.61% | 1094 | 23.75% |
| Hispanic | 604 | 10.30% | 318 | 9.09% | 799 | 17.35% |
| Other | 137 | 2.34% | 94 | 2.69% | 183 | 3.97% |
| Total | 5865 | 100.00% | 3498 | 100.00% | 4606 | 100.00% |
